# Mannose-Decorated Dendritic Polyglycerol Nanocarriers Drive Antiparasitic Drugs To *Leishmania infantum*-Infected Macrophages

**DOI:** 10.3390/pharmaceutics12100915

**Published:** 2020-09-24

**Authors:** Laura I. Vossen, Bárbara Domínguez-Asenjo, Camino Gutiérrez-Corbo, M. Yolanda Pérez-Pertejo, Rafael Balaña-Fouce, Rosa María Reguera, Marcelo Calderón

**Affiliations:** 1 Institute of Chemistry and Biochemistry, Freie Universität Berlin, Takustrasse 3, 14195 Berlin, Germany; livhmio@hotmail.com; 2 Department of Biomedical Sciences, Faculty of Veterinary Medicine, University of León, 24071 León, Spain; bdoma@unileon.es (B.D.-A.); mgutc@unileon.es (C.G.-C.); myperp@unileon.es (M.Y.P.-P.); rbalf@unileon.es (R.B.-F.); 3 POLYMAT & Applied Chemistry Department, Faculty of Chemistry, University of the Basque Country UPV/EHU, Paseo Manuel de Lardizabal 3, 20018 Donostia-San Sebastián, Spain; 4 IKERBASQUE, Basque Foundation for Science, 48013 Bilbao, Spain

**Keywords:** nanocarriers, polyglycerol, amphotericin B, mannosylated surface decoration, macrophage residing *Leishmania infantum*

## Abstract

Macrophages are hosts for intracellular pathogens involved in numerous diseases including leishmaniasis. They express surface receptors that may be exploited for specific drug-targeting. Recently, we developed a PEGylated dendritic polyglycerol-based conjugate (PG–PEG) that colocalizes with intracellular parasite. We hereby study the effect of surface decoration with mannose units on the conjugates’ targeting ability toward leishmania intracellular parasites. Murine and human macrophages were exposed to fluorescently labeled mannosylated PG–PEG and uptake was quantified by flow cytometry analysis. Nanocarriers bearing five mannose units showed the highest uptake, which varied between 30 and 88% in the population in human and murine macrophages, respectively. The uptake was found to be dependent on phagocytosis and pinocytosis (80%), as well as clathrin-mediated endocytosis (79%). Confocal microscopy showed that mannosylated PG–PEGs target acidic compartments in macrophages. In addition, when both murine and human macrophages were infected and treated, colocalization between parasites and mannosylated nanoconjugates was observed. Leishmania-infected bone marrow-derived macrophages (BMM) showed avidity by mannosylated PG–PEG whereas non-infected macrophages rarely accumulated conjugates. Moreover, the antileishmanial activity of Amphotericin B was kept upon conjugation to mannosylated PG–PEG through a pH-labile linker. This study demonstrates that leishmania infected macrophages are selectively targeted by mannosylated PEGylated dendritic conjugates.

## 1. Introduction

Leishmaniasis is a complex of neglected diseases transmitted by the bite of blood-sucking sandflies infected with parasites of the genus Leishmania [1]. Among the different species of *Leishmania*, *L. donovani* and *L. infantum* in the Old World and *L. chagasi (infantum)* in the New World are responsible for the most severe clinical presentation of the disease that can cause the death of the host if left untreated [2]. Hepatosplenomegaly along with renal dysfunction are the most frequent clinical signs of the visceral form of the disease. In addition, post-Kala-azar skin leishmaniasis is a complication associated with *L. donovani* infections in the Indian subcontinent years after the patients were treated with antimony [1,3]. Currently, between 50,000 and 90,000 new cases of visceral leishmaniasis are reported every year, causing between 26,000 and 65,000 deaths, ranking this disease as the second most deadly neglected disease after malaria [4,5].

Antileishmanial pharmacopoeia includes a limited number of drugs, drug combinations and administration protocols, depending on the geographical area and presentation of the disease [1,2,6,7,8]. The antileishmanial drugs currently used include pentavalent antimony derivatives (Sb^V^), the antifungal macrolide amphotericin B (AmB, deoxycholate salt or liposomal formulation), the antibiotic paromomycin and the alkyl-phospholipid (miltefosine), which is the only drug that has good bioavailability after oral administration [9,10,11,12]. Despite the good results shown by these drugs over the years, serious problems have been reported, including poor bioavailability, the emergence of resistant strains, lack of efficacy and high toxicity [13]. In particular, AmB has shown to have good effects against Visceral leishmaniasis, but issues related to nephrotoxicity limit its broader application. Therefore, the introduction of novel drugs or formulations that improve drug bioavailability to accomplish the target product profile (TPP) recommendations made by Drugs for Neglected Diseases initiative for visceral leishmaniasis are urgently needed [14].

In the vertebrate host, *Leishmania* behaves like an intracellular parasite, living and replicating in the acidic environment of macrophage phagolysosomes by subverting the defense mechanisms designed to neutralize them [15] In the course of a natural infection, host macrophages employ more than one receptor to internalize Leishmania parasites, namely: the third and first complement receptors (CR3 and CR1), the mannose receptor (MR), the Fc gamma receptors and the fibronectin receptors (FnRs) [16]. Once phagocytized, promastigotes, which are located within phagosomes, can delay the fusion with lysosomes by a virulence factor called lipophosphoglycan (LPG) that coats the surface of promastigotes [17]. Other receptors such as toll-like receptor 2 or MR also influence phagosome maturation during leishmania infection [18].

In addition to their phagocytic properties, macrophages interact with T lymphocytes and can be classically activated (M1), to give a proinflammatory subtype response that exhibits microbicidal properties or alternatively activated (M2), to yield an anti-inflammatory/regulatory subtype response that is linked to leishmania resistance [19]. Peritoneal macrophages infected with *L. infantum* are characterized by an M2b-like phenotype and C-type lectin receptors signature composed of Dectin-1, mannose receptor (CD206) and the SIGNR3 expression [20]. On the other hand, lesional monocytes and macrophages isolated from patients with post kala-azar dermal leishmaniasis also showed a M2 phenotype with higher expression of CD206 and arginase-1 [21]. CD206 is an endocytic receptor mainly expressed by macrophages and dendritic cells that recognizes sulfated and mannosylated sugars [22]. Recently, it has been widely studied as receptor-targeted for imaging and therapeutic agents against cancer and infectious diseases [23].

The intracellular localization of leishmania amastigotes inside the parasitophorous vacuole hosted by resident macrophages in liver, spleen and bone marrow is a pharmacokinetic concern since the drug must pass through several barriers before reaching sufficient concentrations to kill the parasite [24]. Nanoparticles (NPs) have been considered as a potential solution to deliver antileishmanial drugs inside the phagolysosomal compartment [25]. Previous studies showed that treatment utilizing dendritic polyglycerol decorated with polyethylene glycol (PG–PEG) conjugated to cytotoxic doxorubicin (DOX) increased the antileishmanial effect of the drug, while having moderate cytotoxicity due to controlled drug release inside the parasitophorous vacuole [26]. In the present study, we aim at deciphering the role played by the mannose receptor in the phagocytosis of leishmania parasites, for which we prepared PEGylated dendritic PG drug conjugates that were further decorated with mannose as targeting modality. We examined whether the nanoparticle targeting through the CD206 receptor by mannose surface decoration could increase the potential of AmB against Leishmania parasites. For this, we addressed the cellular uptake and potential colocalization of the mannose-decorated nanocarriers with macrophage residing Leishmania parasites.

## 2. Materials and Methods

### 2.1. Materials

Most of the chemicals and solvents were purchases from Fluka (Buchs, Switzerland), Aldrich (Hamburg, Germany) or Merck (Darmstadt, Germany) and used as received unless otherwise declared. Maleimide-poly(ethylene glycol) (PEG–mal) with MW = 2 kDa and NHS–PEG–alkyne with MW = 3 kDa were purchased from Rapp Polymer (Tübingen, Germany). The 6-Maleimidocaprohydrazide (EMCH) linker was synthesized according to literature [27]. Fluorescein isothiocyanate (FITC), mannose and AmB were purchased from Sigma-Aldrich (Hamburg, Germany). Milli-Q water was prepared using a Millipore water purification system. Purification by centrifugal filtration was performed using Amicon Ultra Centrifugal Filters (molecular weight cutoff, MWCO 3 kDa, Millipore). Size exclusion chromatography (SEC) was performed with Sephadex G-25 superfine (GE Healthcare) under ambient pressure and temperature. Water of Millipore quality (resistivity ~18 MΩ cm^−1^, pH 5.6 ± 0.2) was used in all experiments and for preparation of all samples. If not otherwise specified, sodium phosphate buffer (50 mM) was used for the pH of 7.4, a Britton–Robinson buffer (pH 4.0). All measurements were carried out with freshly prepared solutions at 25 °C. pH values were measured with a Scott instruments HandyLab pH meter at 25 °C.

Phorbol 12-myristate 13-acetate (PMA), trypan blue, thiazolyl blue tetrazolium bromide (MTT), phosphate buffered saline pH 7.4 (PBS) were obtained from Sigma-Aldrich (St. Louis, MO, USA). Wortmannin (W1628), chlorpromazine hydrochloride (C8138), nystatin (N4014) and dextran sulfate sodium salt (D8906) were purchased from Sigma-Aldrich (Deisenhofen, Germany). Dextran was dissolved in dH_2_O and the rest the inhibitors in DMSO to make stock solutions that were further diluted in medium to make their final working concentrations. Transferrin (T13342, Invitrogen) and cholera toxin subunit-b (CTX-B) (C34775, Molecular Probes) both labeled with Alexa Fluor^TM^ 488.

### 2.2. Synthesis of Conjugates

#### 2.2.1. Synthesis of 2-azidoethyl-α-D-mannopyranoside

The three-step procedure (Scheme S1, Supporting Information) was performed according to reported literature [28].

##### Synthesis 2,3,4,6-tetra-O-acetyl-α-D-mannopyranoside

Acetylation of D-mannose (3 g, 17 mmol) was carried out with iodine (150 mg, 0.6 mmol) and acetic anhydride (15 mL, 262 mmol) at 0 °C. After work up and solvent removal under reduced pressure, a yellow viscous liquid (5 g, 75%) at an anomeric mixture (α:β = 4:1) as determined by ^1^H-NMR was obtained (Figure S1).

δ_H_ (400 MHz, CDCl_3_): 1.97 (s, 3H, Ac), 2.01 (s, 3H), 2.05 (s, 3H), 2.13 (s, 3H), 2.14 (s, 3H), 4.00–4.11 (m, 2H), 4.22–4.29 (m, 1H), 5.22–5.34 (m, 3H), 6.04 (d, *J* = 1.92 Hz, 1H).

##### Synthesis of 2-bromoethyl 2,3,4,6-tetra-O-acetyl-α-D-mannopyranoside

1,2,3,4,6-Tetra-O-acetyl-α-D-mannopyranoside (2.4 g, 6.15 mmol) and 2-bromoethanol (0.5 mL, 7.4 mmol) were dissolved under argon in dry DCM (20 mL) and cooled to 0 °C. BF_3_‧Et_2_O (3.9 mL, 30.7 mmol) was added dropwise at 0 °C to the solution. After 1 h of stirring, the reaction mixture was let to reach room temperature (rt) and was kept under stirring overnight. Subsequently, 50 mL of cold water was added, the organic phase was separated, and the aqueous mixture was washed with 50 mL of DCM. The combined organic layers were washed with NaHCO_3_ (50 mL) and water (50 mL). The solution was dried over MgSO_4_ and concentrated under vacuum. The afforded yellow oil was precipitated from diethyl ether, filtered and dried under vacuum to obtain a white solid (1.71 g, 61%).

δ_H_ (400 MHz, CDCl_3_): 1.99 (s, 3H), 2.05 (s, 3H), 2.10 (s, 3H), 2.15 (s, 3H), 3.50–3.53 (m, 2H), 3.85–4.00 (m, 2H), 4.11–4.15 (m, 2H), 4.24–4.29 (m, 1H), 4.87 (d, *J* = 1.62 Hz, 1H), 5.25–5.36 (m, 3H) (Figure S2).

##### Synthesis of 2-azidoethyl 2,3,4,6-tetra-O-acetyl-α-D-mannopyranoside

2-Bromoethyl 2,3,4,6-tetra-O-acetyl-α-D-mannopyranoside (1.1 g, 2 mmol) and sodium azide (0.43 g, 6 mmol) were dissolved in dry DMF (30 mL) under argon and the reaction mixture was stirred at 60 °C for 6 h and afterwards cooled to rt and stirred overnight. Next, the reaction mixture was poured into ethyl acetate (100 mL) and washed with water (2 × 100 mL) and brine (2 × 100 mL). Finally, the combined organic layers were dried over MgSO_4_, filtered and dried under vacuum to afford the product as white crystals (820 mg, 98%).

δ_H_(400 MHz, CDCl_3_): 1.99 (s, 3H), 2.05 (s, 3H), 2.10 (s, 3H), 2.16 (s, 3H), 3.41–3.52 (m, 2H), 3.64–3.89(m, 2H), 4.03–4.13 (m, 2H), 4.26–4.31 (m, 1H), 4.87 (s, 1H), 5.27–5.37 (m, 3H) (Figure S3).

##### Synthesis of 2-azidoethyl-α-D-mannopyranoside (Mannose-N_3_)

Finally, 2-azidoethyl 2,3,4,6-tetra-O-acetyl-α-D-mannopyranoside (500 mg, 1.2 mmol) was deacetylated in a methanolic solution (5 mL) with K_2_CO_3_ (16 mg, 0.12 mmol) at rt overnight. After filtration of remaining K_2_CO_3_ and removing the solvent under vacuum, the product was obtained as a white solid (281 mg, 95%).

δ_H_ (400 MHz, D_2_O): 3.20–3.22 (m, 1H), 3.30–3.33 (m, 2H), 3.45–3.76 (m, 5H, OCH_2_), 3.79–3.85 (m, 1H), 4.72 (d, J = 1.61 Hz, 1H) (Figure S4).

#### 2.2.2. Synthesis of AmB–EMCH

The synthesis of AmB–EMCH was achieved as summarized in Scheme S2. AmB was let to react with 4-acetylbenzoic acid, to yield AmB-bz as previously reported [29]. Subsequently, AmB-bz (50 mg, 0.047 mmol) was dissolved in dry MeOH (0.6 mL) and combined under inert atmosphere (Ar) with EMCH trifluoroacetic acid salt (33.92 mg, 0.099 mmol). The reaction mixture was let to react at rt overnight under stirring, under protection from light. The solution was subsequently concentrated in vacuum and precipitated in ether. The product was centrifuged and washed with ice-cold Et_2_O. The solvent was removed and AmB–EMCH was collected as a yellow solid (55 mg, 0.043 mmol, 92%). The product was characterized by ESI-ToF mass spectrometry and UV-vis spectroscopy (Figure S5).

ESI-TOF: calculated: 1299.6146, found: 1299.6085 [M+Na]^+^.

#### 2.2.3. Synthesis of PG Amine

PG amine was prepared following reported protocols [30] (Scheme S3). In brief, dendritic PG (*M*_n_ ≈ 10 kDa, PDI = 1.6, approximately 135 OH groups) was prepared as previously published [31,32] and then 30% of the total hydroxyl groups (ca. 95 OH and 40 NH_2_ groups per PG scaffold) were converted into amino functionalities in three subsequent steps. These involved partial mesylation of OH groups, followed by substitution of mesyl groups to azide functionalities, which were finally reduced to yield amines. Purification was performed via dialysis after each step and characterization was completed using FT-IR and ^1^H NMR spectroscopy.

#### 2.2.4. Synthesis of PG–PEG–Mann (PEG 3 kDa)

**Scheme 1:**
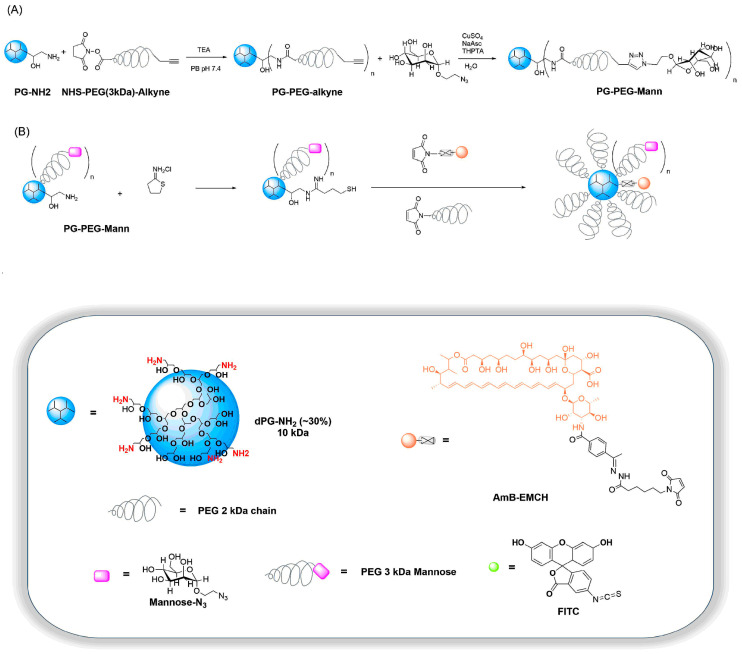
Schematic pathways for the synthesis of (**A**) the precursor PG–PEG–Mann(n) and (**B**) mannose-bearing polymer–drug conjugates. Chemical structures of all the precursors are depicted in the bottom panel.

Mannosylation of PG was performed in two steps as shown in Scheme 1A. Previous to mannose coupling, PG-amine was PEGylated with 5, 10 and 20 equivalents of NHS–PEG–alkyne (MW ≈ 3 kDa) per PG. For this, 20 mg PG amine (*M*_n_ ≈ 10 kDa, 30% amine groups) was dissolved at room temperature in 50 mM sodium phosphate buffer (pH 7.4) and triethylamine (1.13 µL) added to the solution. NHS–PEG–alkyne (5, 10 and 20 equivalents) was added at room temperature with vigorous stirring for two days. The solutions containing PG–PEG–alkyne_5,10 or 20_ were concentrated and washed with Milli-Q water using Centriprep-10-concentrators from Amicon. Afterwards, PEGylated PG-amine was functionalized with mannose in a click reaction. In brief, PG–PEG–alkyne_5,10 or 20_ with free alkyne groups in Milli-Q water reacted with 5, 10 or 20 equivalents 2-azidoethyl-α-D-mannopyranoside (Mannose-N_3_) adding copper sulfate (0.1 eq.), sodium ascorbate (0.2 eq.) and tris(3-hydroxypropyltriazolyl-methyl)amine (0.2 eq.) in MilliQ water to the solution at room temperature. The reaction was stirred at room temperature overnight and the solutions containing PG–PEG–Mann_5,10 or 20_ were concentrated and washed with Milli-Q water using Centriprep-10-concentrators from Amicon and in the end purified by SEC on a Sephadex PD10 column eluting with Milli-Q water. The mannose-loading was determined with an anthrone method as described in Section 2.2.6.

#### 2.2.5. Synthesis of PG–PEG–Mann Drug/Dye Conjugates

**Figure 1:**
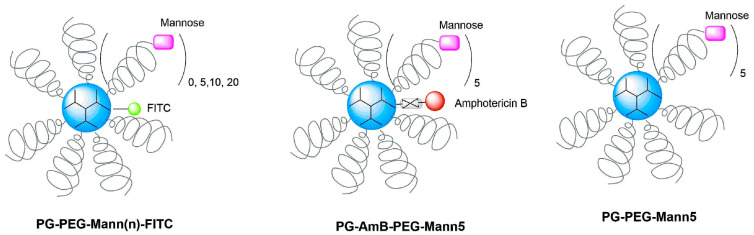
Schematic representation of the PG–PEG–Mann(n) products.

PG–PEG–Mann (with an approximate mannose-loading of 5, 10 and 20 molecules per PG), AmB–EMCH, maleimide-poly(ethylene glycol) (PEG–mal 2 kDa) and FITC were used as building blocks. The amine groups of PG–PEG–Mann were thiolated using 2-iminothiolane as a first step. Michael-type reactions were performed in a sequential manner to conjugate the different maleimide-bearing units to the thiolated PG–PEG–Mann, as described in Scheme 1B. For the reactions where the conjugates are labeled with FITC, the first step comprises a conjugation step without thiolation of the amine groups. Three series of PG–PEG–Mann derivatives were prepared, namely, PG–FITC–PEG–Mann(n), PG–AmB–PEG–Mann5 and PG–PEG–Mann5 (Figure 1).

All reactions were carried out at rt for 20 min under stirring. To several containers with 2 mL of PG–PEG–Mann (6 mg, 0.6 µmol) dissolved in 50 mM sodium phosphate buffer (pH 7.4) 2-iminothiolane (4.9 mg, 36 µmol, 1.5 eq. per NH_2_ group) was added. The products resulting from each step were purified using Sephadex G-25 superfine columns, eluting with 50 mM phosphate buffer (pH 7.4). A solution of AmB–EMCH (1.15 mg/100 µL methanol, 0.9 µmol, 1.5 eq. per PG) was added to the obtained polymeric fractions. After 10 min, a solution of PEG–mal (50 mg/mL, 30 µmol, 50 eq. per PG) in 50 mM sodium phosphate (pH 7.4) was added and the resulting solutions were stirred for 2 h. The solutions were concentrated with Centriprep-10-concentrators from Amicon to a volume of approximately 1 mL. The PG–drug/dye–PEG–Mann derivatives were purified by SEC using a Sephadex G-25 superfine column eluting with 50 mM sodium phosphate buffer (pH 7.4) to give the final conjugates, which were further lyophilized. Ellman’s test, chromatography on reverse phase TLC (70% CH_3_CN/20 mM sodium phosphate pH 7.0) and appearance of a faster band on a Sephadex G-25 column were use as indicative of successful conjugation. The probes for imaging studies PG–PEG–Mann(n)–FITC were synthesized using the same synthetic method, but conjugating FITC to PG–PEG–Mann(n) prior to the PEGylation step. The control probe for the therapeutic efficacy PG–PEG–Mann_5_ was prepared by skipping to add AmB–EMCH to the reaction mixture.

#### 2.2.6. Anthrone Method for Sugar Loading Determination

A calibration curve was obtained with different concentrations of mannose-N_3_ (5, 10, 50, 100 and 200 µg/mL) in MilliQ. The sugar solution was cooled to 0 °C. A 10 mM solution of anthrone reagent in H_2_SO_4_ was also prepared and kept at 0 °C. Afterward, 2 mL of the ice cold anthrone solution were added slowly to the aqueous sugar solution at 0 °C and vortexed. The mixtures were heated to 100 °C for 10 min until a green complex was formed. A calibration curve was achieved by plotting the extinction at 620 nm over the µg sugar.

With the freshly prepared calibration solutions and anthrone solution, the sugar loading in PG was determined. Here, 1 mL of PG–PEG–Mann(n) conjugate solution was cooled to 0 °C and 2 mL of 10 mM ice cold anthrone solution added. The loading was calculated with the equation given by the calibration curve. A schematic summary of the process is depicted in Figure S6.

### 2.3. Physicochemical Characterization of PG–AmB–PEG–Mann Conjugates

^1^H NMR spectra were recorded on a Jeol ECX 400, Bruker AMX 500 or on a Bruker BioSpin AV 700 spectrometer (sample concentration: 5–20 mg in 0.5 mL CDCl_3_ or D_2_O). Chemical shifts are reported in ppm (δ units). For electrospray ionization (ESI) mass spectrometry measurements, a TSQ 7000 (Finnigan Mat) instrument was used. The ESI sample was dissolved in methanol at a concentration of 10–20 µM. Absorption spectra were recorded on a LAMBDA 950 UV-vis/NIR spectrometer (PerkinElmer, USA) using disposable PS cuvettes (minimum filling volume 2.5 mL). Dynamic light scattering (DLS) measurements were performed using a Malvern Zetasizer Nano-ZS 90 (Malvern Instruments), equipped with an integrated He–Ne laser (λ = 633 nm). Prior to the measurements (all done at 25 °C), all samples were filtered through a cellulose acetate (CA) membrane filter with 0.45 μm pore size. The samples were freshly prepared in Milli-Q water at a concentration of 1 mg/mL and were measured under a scattering angle of 173°, using quartz cells.

### 2.4. Drug Release Profile Determination of PG-AmB–PEG Conjugate

The cleavage study of the conjugate PG-AmB–PEG was performed at pH 7.4 and pH 4.0. It was carried out using a Shimadzu Prominence-I LC-2030 liquid chromatography (Shimadzu, Duisburg, Germany) with an internal UV absorption detector (λ = 405 nm) and LabSolutions software. A Hypersil^TM^ ODS C18 column (Thermo Scientific, 100 mm × 4.6 mm, particle size: 5 µm) with a directly connected guard C18 column was employed. Methanol–0.005 M EDTA (90:10) was used as the mobile phase at a flow rate of 1.0 mL min^−1^ under isocratic regime. The injection volume was 25 µL. Stock solutions of AmB in methanol were prepared and assessed by reversed phase HPLC (RP-HPLC) in order to obtain a calibration curve for AmB (0.75–7.5 µg, R = 0.9894) (retention time: 1.1–1.3 min) (Figure S7). The conjugates were dissolved, incubated in different buffer systems (Britton–Robinson buffer of pH 4.0 and sodium phosphate buffer of pH 7.4) and maintained at 37 °C under continuous shaking. Aliquots of 200 µL were taken at specific time intervals (0, 1, 6, 7 and 25 h) and freeze-dried, reconstituted in 200 µL methanol and finally analyzed by RP-HPLC (Shimadzu, Duisburg, Germany).

### 2.5. Mice and Parasites

The animal research described in this manuscript complies with Spanish Act (RD 53/2013) and European Union Legislation (2010/63/UE). The used protocols were approved by the Animal Care Committee of the University of León (Spain), project license number (07/2019). Briefly, six to eight weeks old female BALB/c mice were purchased to Janviers Labs (France) and were housed in specific pathogen-free facilities of University of Leon (Spain) for this study.

The infrared fluorescence-emitting strain iRFP+*L. infantum* was previously described [33]. Promastigotes were routinely cultured at 26 °C in M199 medium supplemented with 25 mM HEPES pH 6.9, 10 mM glutamine, 7.6 mM hemin, 0.1 mM adenosine, 0.01 mM folic acid, RPMI 1640 vitamin mix (Sigma), 10% (*v*/*v*) FBS and antibiotic cocktail (50 U/mL penicillin, 50 mg/mL streptomycin).

### 2.6. Macrophage Cell Cultures and In Vitro Leishmania Infections

Mouse RAW 264.7 macrophages and human THP-1 monocytes were cultured in RPMI medium—high glucose, supplemented with 10% (*v*/*v*) heat inactivated fetal bovine serum (FBS) and 1% (*v*/*v*) antibiotic cocktail (100 U/mL penicillin and 100 mg/mL streptomycin) in a cell incubator at 37 °C with 5.6% CO_2_. Cell lines were kept below passage 20. THP-1 monocytes at 6 × 10^5^ cells/mL were differentiated into macrophages using 200 nM of phorbol 12-myristate 13-acetate (PMA) for 48 h at 37 °C and 5% CO_2_. THP-1 cells were subsequently allowed to fully differentiate in medium without PMA for 5 d, before subjected to experiments [34]. Bone marrow cells were obtained from the femur and tibia of recently sacrificed 10-week old BALB/c female mice and were differentiated to macrophages. Twenty five million cells were cultured at 37 °C for 7 days in 10 mL DMEM medium with L-glutamine (Life Technologies) supplemented with 10% (*v*/*v*) heat-inactivated fetal calf serum (FCS), 10 mM HEPES (pH 7.4), antibiotics (penicillin/streptomycin) and 15% supernatant from cultured L929 cell line as a source of colony-stimulating factor (CSF)-1 [35]. In order to obtain quiescent cells, medium was replaced by free-CSF-1 medium 18 h before further studies.

Macrophages were infected in vitro with freshly iRFP+*L. infantum* amastigotes isolated from infected BALB/c mice spleens. Amastigotes were added to macrophage cultures at an infection ratio of 10:1 and incubated at 37 °C and 5% CO_2_ in complete medium for 12 h. To remove noninternalized parasites, cultures were washed 5-fold with warm PBS and cultured in complete medium at 37 °C in a 5% CO_2_ atmosphere for further studies.

### 2.7. Nanoparticle Uptake Studies

RAW 264.7 macrophages, differentiated THP-1 monocytes and BMM were used for these studies. One-hundred and fifty-thousand RAW 264.7 or PMA-differentiated THP-1 macrophages were cultured for 3 h in the presence of 100 μg/mL mannosylated PG–PEG nanoparticles labeled with FITC. For separation of the internalized and surface-bound nanoparticles, cells were washed 3x with 0.5 M acetate buffer (pH 4.0). FITC-associated fluorescence was measured by flow cytometry (MACS Quant Analyzer 10) at an excitation wavelength of 480 nm and an emission wavelength of 550 nm. At least ten thousand cells were acquired per well. For uptake quantification, side scatter was represented versus FITC fluorescence (FL-1) (x and y axes, respectively). A negative control was established with untreated cells as 2% of the gated population. Data were processed with the FlowJo software. Positive population expressed as percentage (%) was used for uptake quantification.

### 2.8. Subcellular Localization of Nanoparticles

RAW 264.7 or THP-differentiated macrophages were seeded at a density of 6 × 10^4^ cells/mL in 8-well Ibidi (Germany) coated plates. After incubation with 300 μg/mL nanoparticles for 24 h, cells were washed twice with 37 °C prewarmed PBS. Cells were stained with 2.5 µg/mL Hoechst 33342 dissolved in culture medium. Then, 1 μM cresyl violet was also added for 5 min to visualize the acidic compartments [36]. Live macrophages were imaged by using a Zeiss LSM800 confocal microscope Intracellular amastigotes were visualized using a and standard Cy5.5 filter set (665/45 nm exciter and 725/50 nm emitter).

### 2.9. Assessment of Antileishmanial Effect Using Infected ex vivo Splenic Explant Cultures

To determine the comparative antileishmanial effect of free-AmB, AmB-bz and their counterpart conjugated to mannosylated nanoparticles, we used freshly isolated ex vivo splenic explant cultures as before [33]. Briefly, they were cultured in RPMI supplemented with 10% (*v*/*v*) inactivated FBS, 1 mM sodium pyruvate, 1x RPMI vitamins, 10 mM HEPES and antibiotic cocktail (100 U/mL penicillin and 100 mg/mL streptomycin). Starting cell density was fixed at 200,000 counts per 384 clear bottom, black well plate measured at 700 nm in the Odyssey Near-Infrared Fluorescence Imaging System (LI-COR, USA). Free AmB, AmB-bz (which results of acidic hydrolysis of AmB-conjugated nanoparticles) and mannosylated nanoparticles, PG–PEG–Mann5 and PG–AmB–PEG–Mann5 nanoparticles were added at different AmB equivalent concentrations (μM) and incubated at 5% CO_2_ and 37 °C for 96 h. Amastigote viability was assessed by recording the fluorescence emission (700 nm) of infected splenocytes. Vehicle-treated wells (0.4% DMSO) were used as negative controls. Fluorescence measures were used to estimate IC_50_ values using SigmaPlot 10.0 software.

### 2.10. Inhibition of Endocytosis

For inhibition of different endocytic pathways, cells were pre-incubated with three pharmacological pathway inhibitors: chlorpromazine and nystatin for clathrin- and caveolae-mediated endocytosis and the PI-3 kinase inhibitor wortmannin for macropinocytosis and phagocytosis.

Transport of molecules known to selectively enter cells via a specific pathway were used as positive controls to confirm that the required inhibition was achieved, such as transferrin and cholera toxin B for clathrin and caveolae mediated endocytosis, respectively (60 and 0.075 µg/mL for 45 min). A total of 36 × 10^6^ spheres/ mL carboxylate-modified FluoSpheres (1.0 µm) as macropinocytosis and phagocytosis positive control.

## 3. Results

### 3.1. Synthesis and Characterization of Targeting Polyglycerol Conjugates

Dendritic polymers have been widely explored for a series of applications, ranging from electronics to biomaterials. Several dendrimers have been considered in clinical trials, due to their advanced properties, when it comes to structural control at the nanometer scale [37]. Dendritic polyglycerols (PG) of hyperbranched character possess a variety of OH groups (linear and dendritic) that are easy to functionalize, with high control on their loading and position [38,39]. In recent communications, we reported the use of chemical linkers sensitive to changes on the pH or enzyme activity to conjugate prodrugs based on doxorubicin, methotrexate and paclitaxel to PG [40,41,42,43,44]. The modular character of the synthetic strategy that we developed enables the preparation of multifunctional nanocarriers, provided with solubilization enhancers (PEG, for instance), imaging agents and targeting moieties, in a simple procedure [45,46,47,48,49]. We recently used it to prepare PEGylated pH-sensitive PG-doxorubicin conjugates, which showed their potential on murine macrophage cell lines and on *ex vivo* infected BALB/c splenocytes [26]. Although promising, it is expected that such conjugates will not be able to distinguish between healthy and infected cells. Therefore, the current study aims at developing a multifunctional PG system provided with (a) amphotericin B as antileishmania agent or FITC as a fluorescent probe that enables intracellular tracking, (b) PEG as solubilizing and shielding agent and (c) mannose as targeting moiety. Mannose will provide the nanocarrier with targeting ability, in order to decrease toxicity and increase efficacy versus the parasite. It is well stablished that phagolysosomes hosting leishmania parasite have pH values ranging from 4.74 to 5.26 [50]. Therefore, AmB was conjugated to PG through a pH labile hydrazone linker which showed high stability at pH 7.4, with less than 5% of amphotericin released after 24 h incubation, while maximum release occurred at acidic conditions, i.e., 4-fold increase at pH 4.0 (Figure S7). This is the expected behavior for a prodrug system that should release the antileishmania drug only when experiencing an acidic pH at the parasitophorous vacuoles.

A multistep approach was followed, according to Scheme 1, to prepare the different conjugates. The first step (Scheme 1A) comprised the decoration of PG with mannose units: bifunctionalized PEG (NHS and alkyne, at each terminal) with average MW of 3 kDa was first coupled to PG through a peptide linker; this was followed by coupling of azido-bearing mannose to PG–PEG–alkyne, using mannose molar equivalents of 5, 10 and 20. The second step involved the coupling of the further functional moieties, FITC or AmB–EMCH and PEG-Mal of average MW of 2 kDa. For the coupling of AmB–EMCH and PEG–mal, a thiolation was performed on the remaining amino groups of PG, using 2-iminothiolane. Conjugate formation was confirmed by Ellman’s test, chromatography on reverse phase TLC and appearance of a faster band on a Sephadex G-25 column. Figure 1 shows schematic representations from the PG–PEG–Mann systems that were prepared.

AmB loading in each conjugate was determined photometrically at 405 nm (ε_495_ = 25,358 M^−1^ cm^−1^) after reconstitution of the lyophilized samples with sodium phosphate buffer pH 7.4. The characteristics of the polymer conjugate precursors and products are presented in Table 1.

Hydrodynamic diameters of all products were determined by DLS, showing sizes between 12 and 28 nm. PG–PEG–FITC showed to have the smallest diameter (11.91 nm) while PG–PEG–Mann10–FITC, the largest (28.00 nm). The sizes of PG–PEG–Mann5–FITC, PG–PEG–Mann20–FITC and PG–AmB–PEG–Mann5 ranged between 15 and 20 nm (19.95 nm versus 19.46 nm versus 15.05 nm, respectively). These values result from the analysis of the size distribution measurements by volume, as all products had some aggregation and relatively high PDIs (0.3–0.7) (Table S1). More parameters, such as the z-average and size distribution by intensity are summarized in Table S1. Figure S8 shows the DLS graphs with size distributions by intensity and volume.

### 3.2. Internalization of Mannosylated PG–PEG Nanocarrier in Macrophages Depends on CD206 Receptor

**Figure 2:**
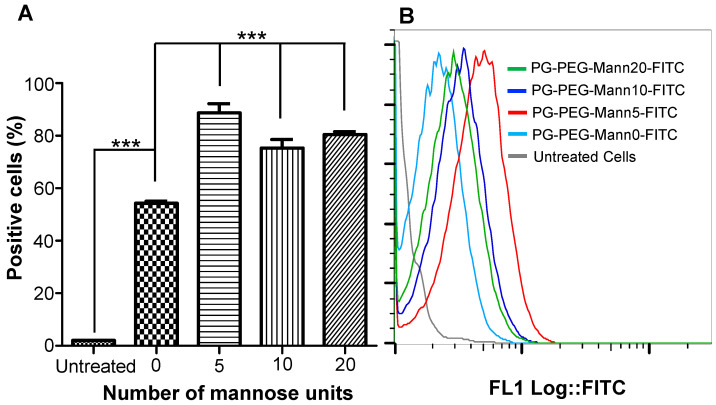
Cellular uptake of mannosylated PG–PEG nanocarrier. (**A**) PG–PEG–Mann(n)–FITC with n equal to 0, 5, 10 and 20 mannoses were incubated with RAW 264.7 macrophages for 24 h. Macrophages were harvested, and the unbound polymers were removed by acidic treatment. FITC fluorescence intensity was acquired on logarithmic scale and represented versus scatter side. Gate FITC-positive population was established as 2% in untreated cells. Uptake is expressed as %. Values are means ± SD; (**B**) flow cytometry histogram for uptake studies in RAW 264.7 macrophages. X-axis represents fluorescence inside the cells. Control untreated cells (gray), PG–PEG–Mann0–FITC (cyan blue), PG–PEG–Mann5–FITC (red), PG–PEG–Mann10–FITC (dark blue), PG–PEG–Mann20–FITC (green). Black asterisks indicate P values for *t*-Student’s test (**A**). Comparisons between untreated and PG–PEG–Mann0–FITC (*** *P* < 0.001) and this later and nanoparticles with 5, 10 and 20 mannose moieties.

Cellular uptake of mannosylated nanocarriers was quantitatively investigated by flow cytometry. The amount of each sample internalized by cells should be proportional to the fluorescence intensity of FITC. Taken into account the amount of drug released after 24 h at pH 4.0; a fixed quantity of 100 μg/mL of each nanoparticle (PG–PEG–FITC) bound to different mannose units (0, 5, 10 and 20), was added to one-hundred and fifty thousand RAW 264.7 murine macrophages and incubated at 37 °C for 24 h. The incubation time for this cell line was optimized in a previous report by the authors, using similar type of nanocarriers [26]. All mannosylated PG–PEG–FITC nanocarriers included in this study were significatively internalized by RAW 264.7 cultures within the first 24 h. Functionalization with only five mannose units (PG–PEG–Mann5–FITC) showed the maximum significant uptake. In addition, 10 and 20-mannose decorated nanocarriers have also shown significant uptake (Figure 2A). Figure 2B displays a representative flow cytometry histogram where the MFI illustrates fluorescence inside cells. These results are consistent with previous reports demonstrating the role played by mannose density on nanoparticle uptake by binding to mannose receptor CD206 externally displayed by macrophage [51,52,53].

**Figure 3:**
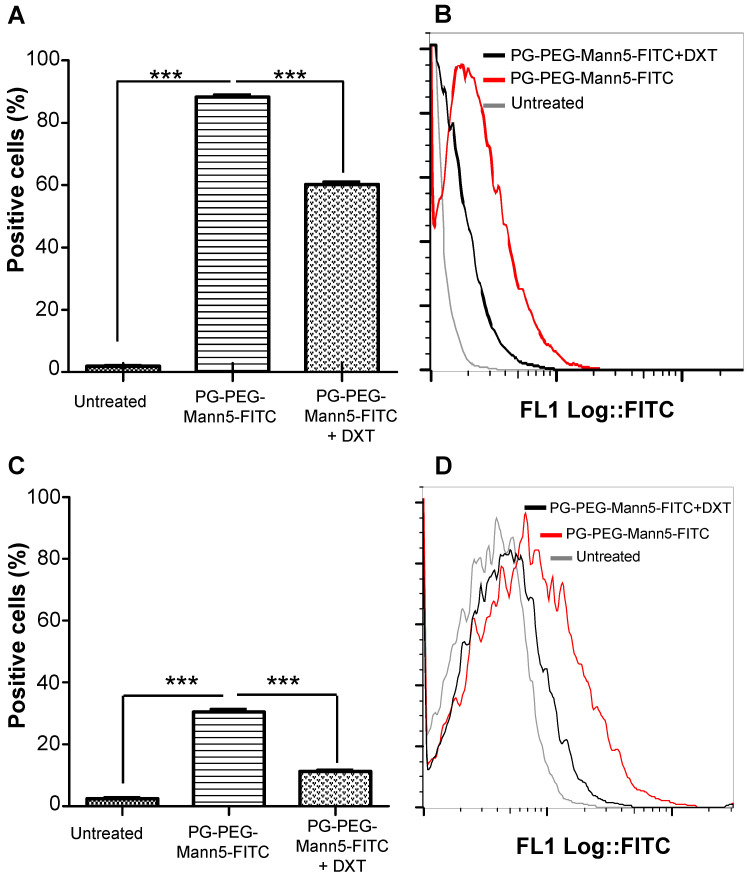
Dextran competition for CD206 receptor decreases the mannose decorated PG–PEG–Mann5–FITC nanoparticle uptake in murine macrophages RAW 264.7 and human THP-differentiated macrophages. (**A**) RAW 264.7 macrophages and (**C**) human THP-differentiated macrophages were preincubated for 30 min with or without 5 mg/mL of dextran before the addition of mannosylated nanocarriers. After 24 h incubation at 37 °C with nanoparticle, uptake was quantified by flow cytometry. Uptake is expressed as%. Values are means ± SD. Histograms show the Mean Fluorescent Intensity MFI that represents fluorescence inside the cells; (**B**) RAW 264.7 macrophages and (**D**) human THP-differentiated macrophages. Control untreated cells (gray), PG–PEG–Mann5–FITC without dextran (red), PG–PEG–Mann5–FITC with dextran (black). Black asterisks indicate P values for *t*-Student’s test (**A**,**C**). Comparisons between PG–PEG–Mann5–FITC in dextran-free or dextran containing medium (*** *p* < 0.001).

In order to know whether the internalization of mannosylated nanocarriers was dependent on CD206 receptor, RAW 264.7 macrophages were preincubated for 30 min with 5 mg/mL dextran (DXT), a demonstrated competitor of CD206 [54]. PG–PEG–Mann5–FITC nanoparticle was added to cultures as above. Figure 3A,B show much greater cellular uptake of the nanoparticle in the dextran-free (88.4%) than in the dextran-containing medium (60.35%). Based on these results, the presence of dextran in the medium prevented PG–PEG–Mann5–FITC from entering into the RAW 264.7 macrophages by competitively binding to the CD206 receptor on cell surface, indicating that PG–PEG–Mann5–FITC was mostly internalized through mannose receptor-mediated endocytosis.

In order to assess whether this phenomenon was exclusive of murine RAW 264.7 cells or a general process common to other macrophagic cell lines, we studied the uptake of PG–PEG–Mann5–FITC in human THP-differentiated macrophages. First, we need to establish the time for nanoparticle entry in this cell line. Figure S9 illustrates the accumulation of FITC into THP-differentiated macrophages incubated at 37 °C, as revealed by flow cytometry. Since PG–PEG–Mann5–FITC uptake was detected at the first 24 h, we choose that time to incubate THP-differentiated macrophages in the presence or absence of dextran. Figure 3C shows a decrease in the PG–PEG–Mann5–FITC uptake (from 30.85% to 11.5%) when cells were pretreated with dextran, suggesting that the entrance of mannosylated nanoparticle in THP-differentiated macrophages could be through CD206 receptor. Figure 3D shows a representative histogram that correspond to data in Figure 3C.

However, since dextran was not able to fully abolish PG–PEG–Mann5–FITC uptake in neither of the tested macrophage cultures, we studied whether other endocytic pathways mechanisms could be involved. RAW 264.7 macrophages were preincubated for 30 min with three pharmacological pathway inhibitors: 100 µM chlorpromazine, 160 µM nystatin and 30 μM wortmannin. As positive controls, we used transferrin (TfR) and cholera toxin subunit-b (CTX-B) labeled with Alexa Fluor™ 488 to confirm the inhibition of clathrin- and caveolin-mediated endocytosis, respectively upon treatment with chlorpromazine [55] and nystatin [56]. Carboxylate-modified FluoSpheres^TM^ (1.0 µm) beads labeled with yellow-green fluorescent dye were used as control to confirm the inhibition of macropinocytosis and phagocytosis by wortmannin [57].

**Figure 4:**
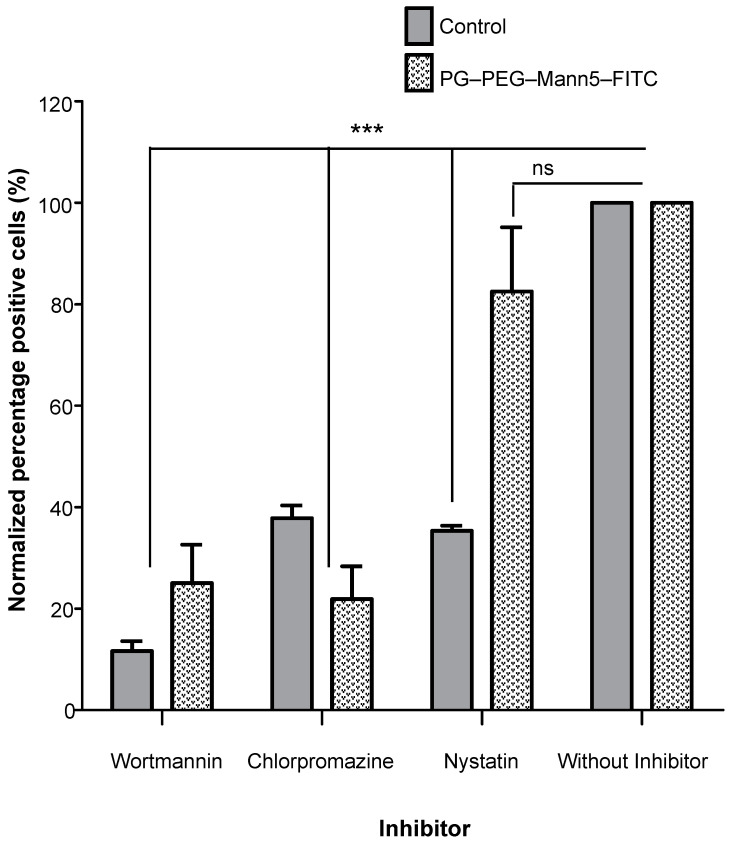
Effects of different pharmacological inhibitors on the uptake of PG–PEG–Mann5–FITC. RAW 264.7 macrophages were preincubated for different times with inhibitors, treated with PG–PEGMann5–FITC for 24 h and then fixed and analyzed by flow cytometry. Results are reported as the percentage of uptake relative to untreated cells exposed to NPs (set to 100%). As positive controls were used FluoSphere (1.0 µm), Transferrin and cholera toxin subunit-B (gray boxes). Values are expressed as normalized percentage of positive cells ± SD of three independent duplicate experiments. Black asterisks indicate *p* values for *t*-Student’s test. Comparisons between cells treated with endocytic inhibitors and untreated cells (*** *p* < 0.001).

Figure 4 shows that in RAW 264.7 macrophages, the inhibition of macropinocytosis and phagocytosis by wortmannin led to 74.94% reduction on the uptake of PG–PEG–Mann5–FITC. Likely, the inhibition of clathrin-mediated endocytosis by chlorpromazine led to 78.08% reduction on the nanoparticle uptake. In both endocytic pathways, positive controls with beads and transferrin confirmed the applied inhibition protocol with 90.38% and 64.68% reduction, respectively.

However, nystatin only reduced 17.51% the nanoparticle uptake, despite the CTX-B control showed 65.62% reduction. These results highlight the complexity of mechanisms to endocytose NPs and suggest that PG–PEG–Mann5–FITC may be internalized via different pathways in macrophages. The data presented here strongly suggest the dependence on phagocytosis and micropinocytosis as well as clathrin-mediated endocytosis, while caveolin-mediated endocytosis played a lesser role. None of the pharmacological treatments could fully inhibit NP uptake and this leaves open the possibility that multiple pathways can be simultaneously used to internalize a nanoparticle. Recently, caveolin-mediated phagocytosis of *L. donovani* has been proposed as pathway to gain access to the host cell interior [58], consequently, PG–PEG–Mann5–FITC would not interference with this pathway of parasite. In addition, the small fraction of aggregates found by DLS for PG–PEG–Mann5–FITC may be responsible for the uptake found through phagocytosis.

### 3.3. L. infantum Infected Macrophages Display Higher Avidity for Mannosylated Nanocarriers than Non-Infected Macrophages

**Figure 5:**
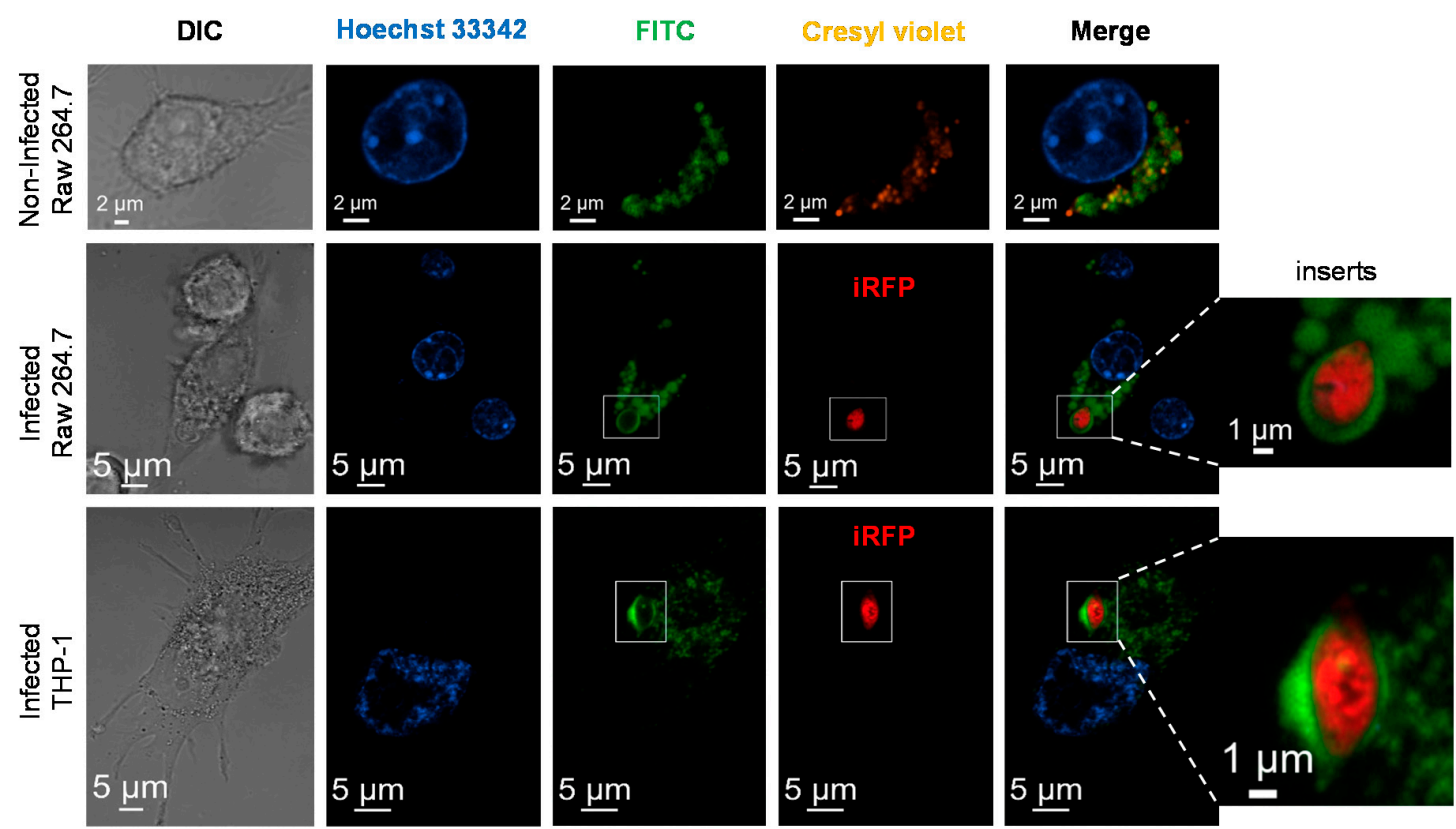
Mannosylated nanocarriers reach the acidic compartments of macrophages and colocalize with *L. infantum* parasites in phagolysosomes. (**top**) Confocal micrographs of uninfected RAW 264.7 macrophages treated for 24 h with PG–PEG–Mann5–FITC, stained with Hoechst 34580 and cresyl violet and visualized in vivo; (**middle**) confocal micrographs of *L. infantum*-infected RAW 264.7 macrophages treated as above; (**bottom**) confocal micrographs of *L. infantum*-infected THP-1-differentiated macrophages. White scale bars in the bright field microscopy pictures are 5 μm and 1 mm in the inserts where compartments with a clear localization between nanoparticle (FITC) and *L. infantum* amastigotes (red) is shown.

On the grounds that leishmania are intracellular parasites and reside inside the parasitophorous vacuole, we next ask whether the mannosylated conjugates were able to use the endocytic pathway to reach the phagolysosome and hence the intracellular parasite. Uninfected RAW 264.7 macrophages were incubated 24 h with 300 μg/mL PG–PEG–Mann5–FITC, before the lysosomes and nuclei were stained using cresyl violet and Hoechst 34580. Cells were analyzed by live cell imaging using a confocal microscope. Confocal micrographs are shown in Figure 5.

PG–PEG–Mann5–FITC was accumulated in well-defined vacuoles (Figure 5 top panel), some of them surrounded by LAMP (data not shown), displaying their accumulation in late endosomes and lysosomes. When a lysosomal marker as cresyl violet (orange) was used, it overlapped with some of the organelles that contained FITC (as evident from the overlay of the green and red channels in the merge column). These results suggest that although PG–PEG–Mann5–FITC is present in different endosomes, it may accumulate in late endosomes and lysosomes of the macrophages. Such localization is in agreement with previously obtained results using the same kind of nanoparticle without mannose decoration [26].

Although lysosomal compartment has been considered as a homogeneous chamber for long, it has recently been reported that peripheral regarding perinuclear lysosomes are less acidic in a single and intact cell [59]. In addition, FITC has its maximum emission wavelength sensitive to the pH, hence its fluorescence decreases at acidic pH. On the contrary, cresyl violet increases its fluorescence at acidic environments, which may justify that not all the colocalization points are yellow as expected.

Given the intracellular localization of mannosylated PG–PEG nanocarriers into late endosomes and lysosomes, it is possible that it colocalizes with *L. infantum* amastigotes in the parasitophorous vacuole and, hence, be a potential drug carrier for treatment of such pathogen caused diseases. Figure 5 middle panel demonstrates that the green fluorescence emitted by PG–PEGMann5–FITC colocalized with iRFP-*L. infantum* amastigotes (red) in RAW 264.7 macrophages. Zooming of the boxed area (insert) shows RAW 264.7 macrophages infected with *L. infantum* amastigotes (red) upon 12 h incubation with PG–PEG–Mann5–FITC nanoparticle (green). Insert shows a green halo (corresponding to FITC) surrounding the intracellular amastigote (red), suggesting that PG–PEG–Mann5–FITC nanoparticles reached the phagolysosomes. In order to confirm these data, we also infected human THP-1 differentiated macrophages. Bottom panel in Figure 5 shows confocal micrographs where phagolysosomes housing iRFP-*L. infantum* amastigotes (red) were surrounded by PG–PEG–Mann5–FITC nanoparticles (green). Collectively, these data confirm that mannosylated PG–PEG nanocarriers accumulated in lysosomes in non-infected macrophages and colocalized with intracellular *L. infantum* amastigotes in infected macrophages. These results leave open the possibility that drugs can be preferentially driven to phagolysosomes, a secure niche where intracellular pathogens can proliferate and therefore this sanctuary could end when drugs reach those compartments.

However, not all tissue-resident macrophages in an organ remain infected, thus designing nanocarriers that recognized specifically infected cells are crucial. In order to quantify the colocalization between parasites and mannosylated nanocarriers in infected versus uninfected macrophages, we performed flow cytometry analysis. BMM were infected or no with *iRFP-L. infantum* amastigotes isolated from infected BALB/c mice spleens. Upon removal of non-internalized parasites, cultures were treated with PG–PEG–Mann5–FITC nanoparticle and flow cytometry analysis was performed in order to know the nanoparticle preference for infected versus non-infected BMM. Infrared fluorescence from intracellular parasites was detected with APC channel in MACs QuaNT 10 (655–730 nm)

**Figure 6:**
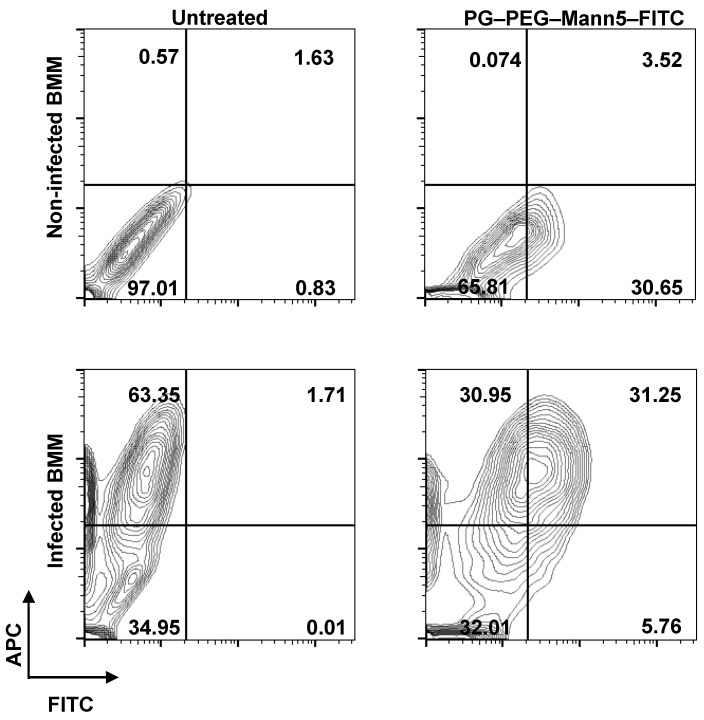
Quantification iRFP-*L. infantum* intracellular amastigotes (FL7) and PG–PEG–Mann5–FITC (FL3) colocalization. Bone marrow derived macrophages were infected with iRFP-*L. infantum* amastigotes. Then, uninfected and infected cultures were treated or non-treated with PG–PEGMann5–FITC nanoparticle. (**top**) Non infected bone marrow derived macrophages; (bottom) iRFP-*L. infantum*-infected bone marrow derived macrophages; (**right**) cultures untreated; (**left**) cultures treated with PG–PEGMann5–FITC for 24 h before cells analyzed by flow cytometry.

Right top panel in Figure 6 shows that 33% (30.65 + 3.52) of uninfected macrophages were positive for FITC, meaning that one-third of population have internalized the mannosylated-NPs. When the experiment was performed with iRFP-*L. infantum*-infected macrophage cultures, the colocalization of NP with fluorescent parasites allow us to know how the distribution of NP between the population is, since not all macrophages in the well culture are infected. Left bottom panel in Figure 6 shows that 63% of macrophages were positive for allophycocyanin (APC), meaning that they were infected, versus 34% uninfected macrophages (negative for APC). The treatment for 24 h with PG–PEG–Mann5–FITC, led to half of infected macrophages (31.25/63.35) internalized the NP (31.25% positive for APC and FITC), whereas the internalized NP by uninfected macrophages was only 5.76% (negative for APC and positive for FITC), which represented 16% population (5.76/34.95). Collectively, these results confirm that *L. infantum*-infected macrophages have higher avidity for mannosylated nanocarriers than non-infected macrophages. Our outcomes agree with recently results where bivalent mannosylated nanocarriers have been demonstrated to utilize the CD206 receptor as portal for entry in alternative differentiated macrophages or M2 [60]. This phenotype could be induced either by IL-4/IL-13 macrophages treatment or leishmania infection, which would justify the use of mannosylated carriers for drug delivery in leishmania. Macrophage polarization from M1 to M2 was described in other intracellular pathogens such as *Mycobacterium tuberculosis* during the formation and development of tuberculous granulomas [61], *Candida albicans* [62] and *Salmonella typhimurium* [63].

### 3.4. Mannosylated Nanoparticles as Drug Carriers

We first studied the biocompatibility of the mannosylated conjugates in the murine macrophage cell line RAW 264.7. Cells were exposed to 300 μg/mL PG–PEG–Mann5 nanoparticle for 96 h at 37 °C. No cytotoxicity on macrophage cultures was found at this concentration of NP (data not shown).

In order to assess the antileishmanial effect of these nanocarriers, we incubated freshly ex vivo splenic explants isolated from 6 weeks iRFP-*L. infantum*-infected BALB/c mice with free-AmB, AmB-bz, PG–AmB–PEG–Mann5 or PG–PEG–Mann5. Free-AmB and AmB-bz (product resulting of the acid hydrolysis of AmB with the nanoparticle) were used as positive controls while PG–PEG–Mann5 was used as negative control.

**Figure 7:**
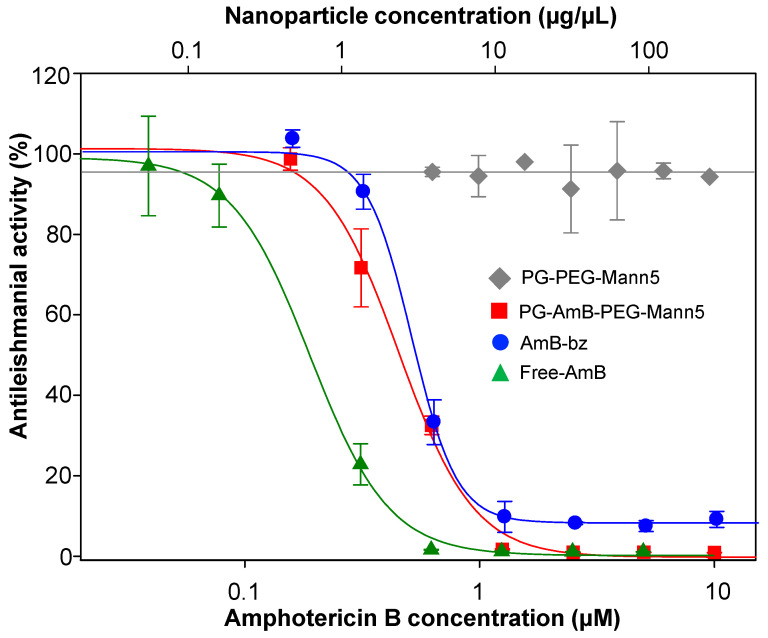
Curve dose–response of free and conjugated drugs to PG–PEG–Mann5 nanoparticle against intracellular iRFP-*L. infantum.* Ex vivo splenic explants were incubated at 37 °C for 96 h with different equivalent concentrations of free-AmB (green), AmB-bz (blue), PG–AmB–PEG–Mann5 (red) and PG–PEG–Mann5 (gray).

Vehicle-treated wells (0.4% DMSO) and 300 μg/mL PG–PEG–Mann5 were used as negative controls. After 96 h incubation, we used an Odyssey imaging system (Li-Cor) to obtain infrared fluorescence measures to calculate IC_50_ values by using SigmaPlot 10.0 software. Figure 7 shows that both AmB-bz and PG–AmB–PEG–Mann5 had similar IC_50_ values (0.43 ± 0.03 µM and 0.48 ± 0.01 µM, respectively), which was twice the IC_50_ value for free-AmB (0.20 ± 0.02 µM), while the empty nanoparticle PG–PEG–Mann5 was inactive. Both, AmB-bz (the ester-derivative of AmB at the glucosamine position) and PG–AmB–PEG–Mann5 showed slightly lower antileishmanial activity than free-AmB. However, as we stated above, PG–AmB–PEG–Mann5 would be preferentially internalized by infected cells which would reduce the toxicity of AmB in other non-infected cells. Despite the current efforts done by pharma industry and academia in searching for better treatments against leishmaniasis, most of candidates drop the race due to the peculiar scenario displayed by this disease (parasites reside in intracellular organelle and multiple organs are affected), which make even more difficult to address issues as toxicity and biodistribution [64].

Alternative activation pathway expressed by macrophages is present in several pathologic conditions. Leishmaniasis is a chronic disease with parasites mainly hosted in macrophages of different organs and tissues. Infected macrophages are activated though the alternative pathway, which avoids an adequate immune response thus, allowing parasites replication inside the phagolysosome. To target specifically through alternatively activated macrophages instead of just macrophages would favor a more specific delivery of drugs in many pathologies. Previously, we reported a dendritic nanoparticle that target macrophages in general. In this study, we have decorated the same nanoparticle with mannose moieties on surface. This mannosylated nanoparticle targeted to CD206 receptor has shown higher accumulation in leishmania infected macrophages versus non-infected macrophages. This could be a new approach to be explored in those fields that alternative macrophage activation is responsible for pathology, including Leishmaniasis. In addition, there are available in vivo models of this disease that make feasible nanoparticle in vivo investigation [65].

## 4. Conclusions

We synthesized and characterized mannosylated PEGylated dendritic PG conjugates in which additionally AmB was attached via a pH-cleavable hydrazone bond. The release of the drug at acidic pH was demonstrated and the mannose-loading was determined via the anthrone method. With this study, we developed nanoconjugates that are able to reach the phagolysosome in leishmania-infected macrophages. The idea of decorating the nanoconjugates surface with mannose units was based on the previous knowledge of the M2-macrophage phenotype expressed on experimental and human visceral leishmaniasis, where the endocytic receptor CD206 increases its expression on macrophages and dendritic cells. These new mannose-decorated dendritic nanocarriers have shown to be endocytosed through different pathways and preferably taken up by macrophages infected by leishmania rather than non-infected macrophages. Thus, these nanocarriers represent a promising specific drug-delivery vehicle against leishmaniasis.

## Figures and Tables

**Table 1:** Summary of characteristics of precursors and products.

Compound	MW of PG Core ^a^	Reactive Groups per PG ^b^	AmB Loading in weight% ^c^	FITC Loading in Weight% ^d^	Mannose Loading in Weight% ^e^
PG–PEG–FITC	10 kDa	40 NH_2_	*–*	0.23	*–*
PG–PEG–Mann5–FITC	10 kDa	40 NH_2_	*–*	0.29	0.77
PG–PEG–Mann10–FITC	10 kDa	40 NH_2_	*–*	0.29	1.66
PG–PEG–Mann20–FITC	10 kDa	40 NH_2_	*–*	0.29	4.71
PG–PEG–Mann5	10 kDa	40 NH_2_	*–*	*–*	0.83
PG–AmB–PEG–Mann5	10 kDa	40 NH_2_	0.43	*–*	0.41

^a^ Determined by GPC including MALS detector (PDI = 1.6); ^b^ determined by ^1^H-NMR spectroscopy; ^c^ determined by UV/Vis spectrometry and absorption coefficient of AmB (ε_405_ = 25,358 M^−1^ cm^−1^); ^d^ determined by UV/Vis spectrometry and absorption coefficient of FITC (ε_500_ = 80,000 M^−1^ cm^−1^); ^e^ determined by UV-vis spectrometry with anthrone at 620 nm.

## Supplementary Materials

The following are available online at https://www.mdpi.com/1999-4923/12/10/915/s1. Table S1: Physicochemical Characterization of PG-PEG-Mann(n)-FITC conjugates; Scheme S1: Synthesis of 2-azidoethyl-α-D-mannopyranoside and reaction yields of each step; Figure S1: 1H-NMR spectrum of 2,3,4,6-tetra-O-acetyl-α-D-mannopyranoside; Figure S2: 1H-NMR spectrum of 2-bromoethyl 2,3,4,6-tetra-O-acetyl-α-D-mannopyranoside; Figure S3: 1H-NMR spectrum of 2-azidoethyl 2,3,4,6-tetra-O-acetyl-α-D-mannopyranoside; Scheme S2. Synthesis of AmB-EMCH; Figure S5: Calibration curve of AmB-EMCH in methanol measured by UV/Vis at 405 nm; Scheme S3: Synthesis of amine-bearing polyglycerol; Figure S6: Quantification of mannose loading via Anthrone method; Figure S7: (A) Representative release profile of PG-AmB-PEG incubated at pH 4.0 and 7.4 at 37 °C over 25 h. The AmB release (%) was quantified by RP-HPLC. Mean ± SEM were obtained from triplicates in three independent experiments. (B) Calibration curve for AmB in methanol measured by RP-HPLC at a retention time of 1.1 min with methanol-0.005M EDTA (90:10) as mobile phase at a flow rate of 1.0 mL min-1 under isocratic regime. The injection volume was 25 μL. Figure S8: Dynamic light scattering measurement showing the size distribution by volume of conjugate (A) PG-PEG-FITC, (B) PG-PEG-Mann5-FITC, (C) PG-PEG-Mann10-FITC, (D) PG-PEG-Mann20-FITC and (E) PG-AmB-PEG-Mann5; Figure S9. Cellular uptake kinetic profile obtained by flow cytometry for PG-PEG-Mann5-FITC.
