# Supplementary material for: Mannose-Decorated Dendritic Polyglycerol Nanocarriers Drive Antiparasitic Drugs To Leishmania infantum-Infected Macrophages
Source: Pharmaceutics. 2020 Sep 24;12(10):915. doi: 10.3390/pharmaceutics12100915 (PMC7598597; doi:10.3390/pharmaceutics12100915)
Supplement: Supplementary file 1 [file pharmaceutics-12-00915-s001.pdf]

pharmaceutics

Supplementary Materials: Mannose-Decorated Dendritic Polyglycerol Nanocarriers Drive Antiparasitic Drugs To *Leishmania infantum*-Infected Macrophages

Laura I. Vossen [1,†], Bárbara Domínguez-Asenjo [2,†], Camino Gutiérrez-Corbo [2], M. Yolanda Pérez-Pertejo [2], Rafael Balaña-Fouce [2], Rosa María Reguera [2,*], Marcelo Calderón [3,4,*]

1 Institute of Chemistry and Biochemistry, Freie Universität Berlin, Takustrasse 3, 14195 Berlin, Germany; livhmio@hotmail.com
2 Department of Biomedical Sciences, Faculty of Veterinary Medicine, University of León, 24071 León, Spain; bdoma@unileon.es (B.D.-A.); mgutc@unileon.es (C.G.-C.); myperp@unileon.es (M.Y.P.-P.); rbalf@unileon.es (R.B.-F.)
3 POLYMAT & Applied Chemistry Department, Faculty of Chemistry, University of the Basque Country UPV/EHU, Paseo Manuel de Lardizabal 3, 20018; Donostia-San Sebastián, Spain
4 IKERBASQUE, Basque Foundation for Science, 48013 Bilbao, Spain
* Correspondence: rmregt@unileon.es (R.M.R.); marcelo.calderon@polymat.eu (M.C.); Tel.: +34-987295225 (R.M.R.); +34-943018182 (M.C.)
† These authors contributed equally to the article

Received: 3 July 2020; Accepted: 21 September 2020; Published: date

Table S1. Physicochemical Characterization of PG-PEG-Mann(n)-FITC conjugates.

Compound	Z-Average [nm]	Size d (Intensity) [nm]	Size d (Volume) [nm]	PDI
**PG-PEG-FITC**	20.36	16.15	11.91	0.496
**PG-PEG-Mann5-FITC**	41.22	247.1	19.95	0.748
**PG-PEG-Mann10-FITC**	71.96	125.9	28.00	0.448
**PG-PEG-Mann20-FITC**	35.72	60.62	19.46	0.399
**PG-AmB-PEG-Mann5**	28.99	18.22	15.05	0.359

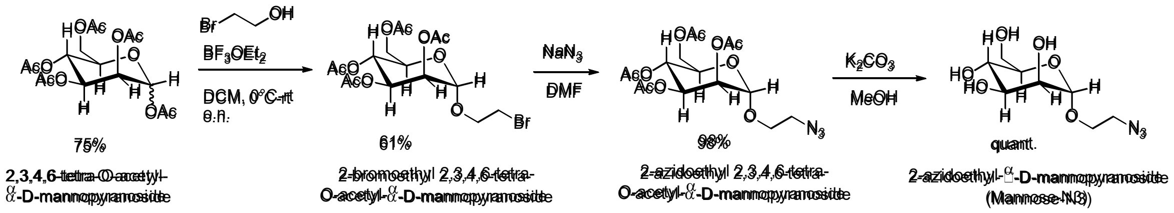

Scheme S1. Synthesis of 2-azidoethyl-α-D-mannopyranoside and reaction yields of each step.

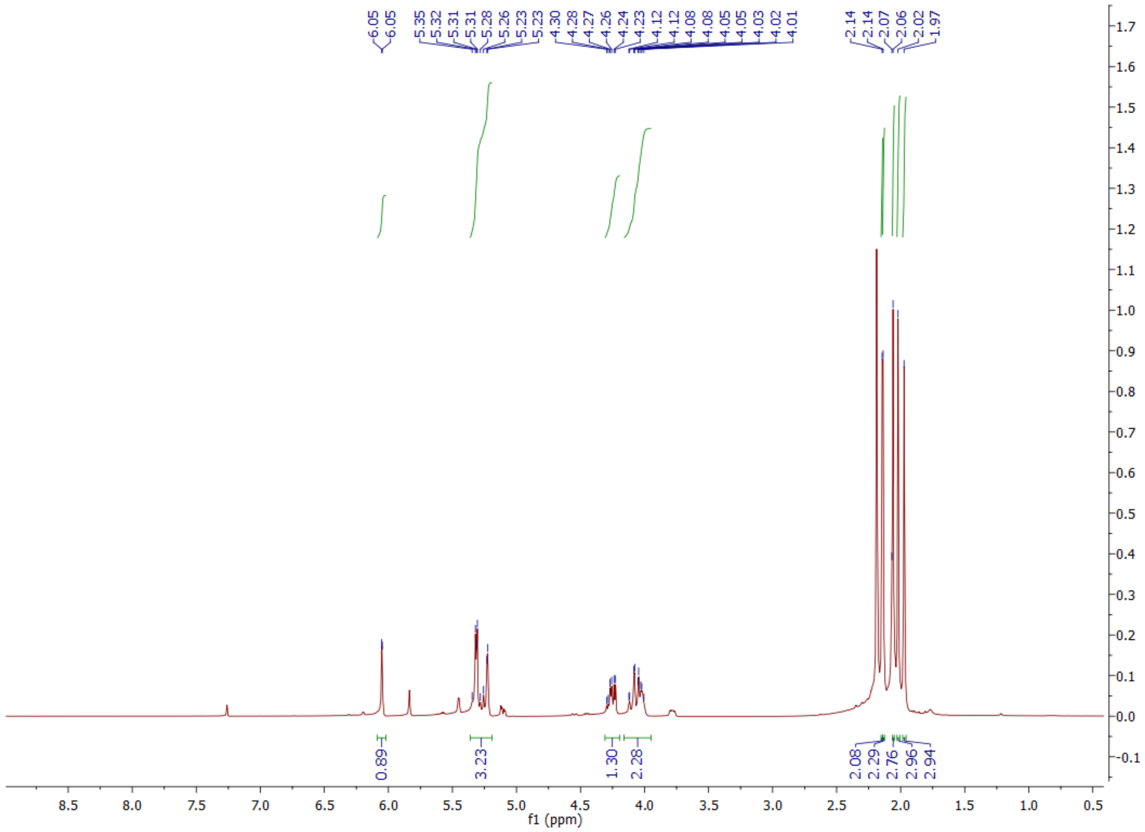

Figure S1. ^{1}H-NMR spectrum of 2,3,4,6-tetra-O-acetyl-α-D-mannopyranoside.

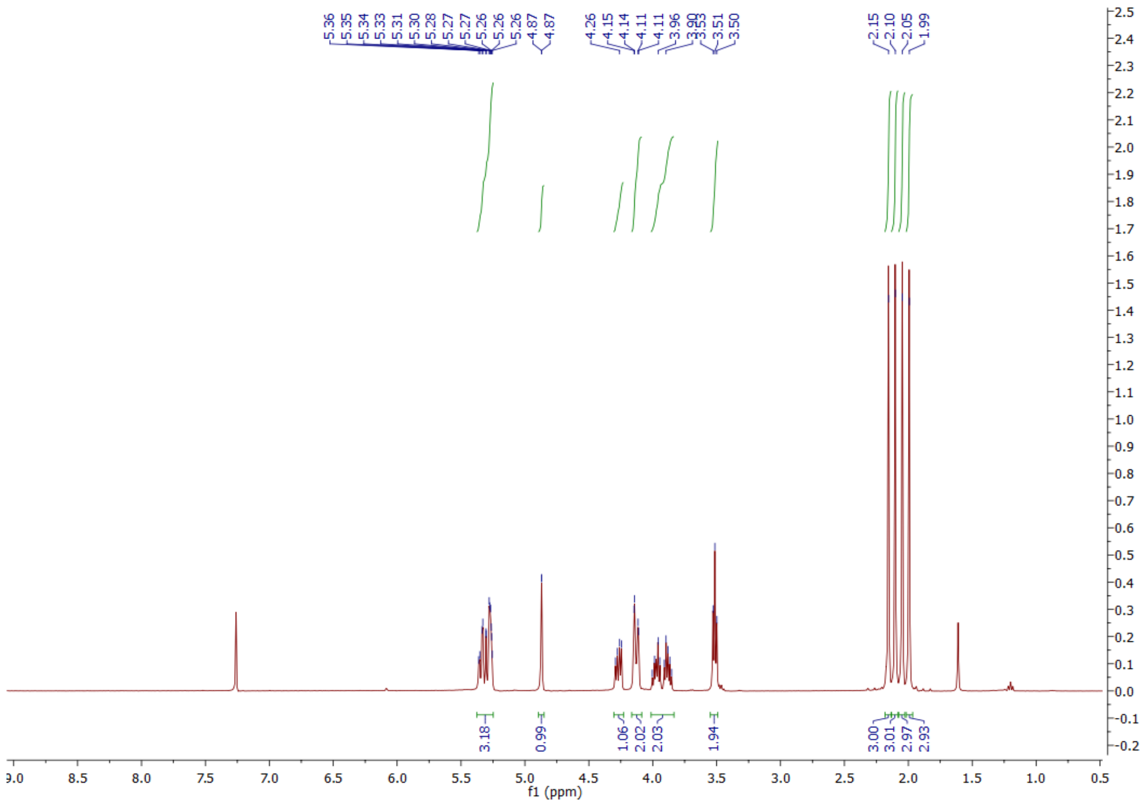

Figure S2. ^{1}H-NMR spectrum of 2-bromoethyl 2,3,4,6-tetra-O-acetyl-α-D-mannopyranoside.

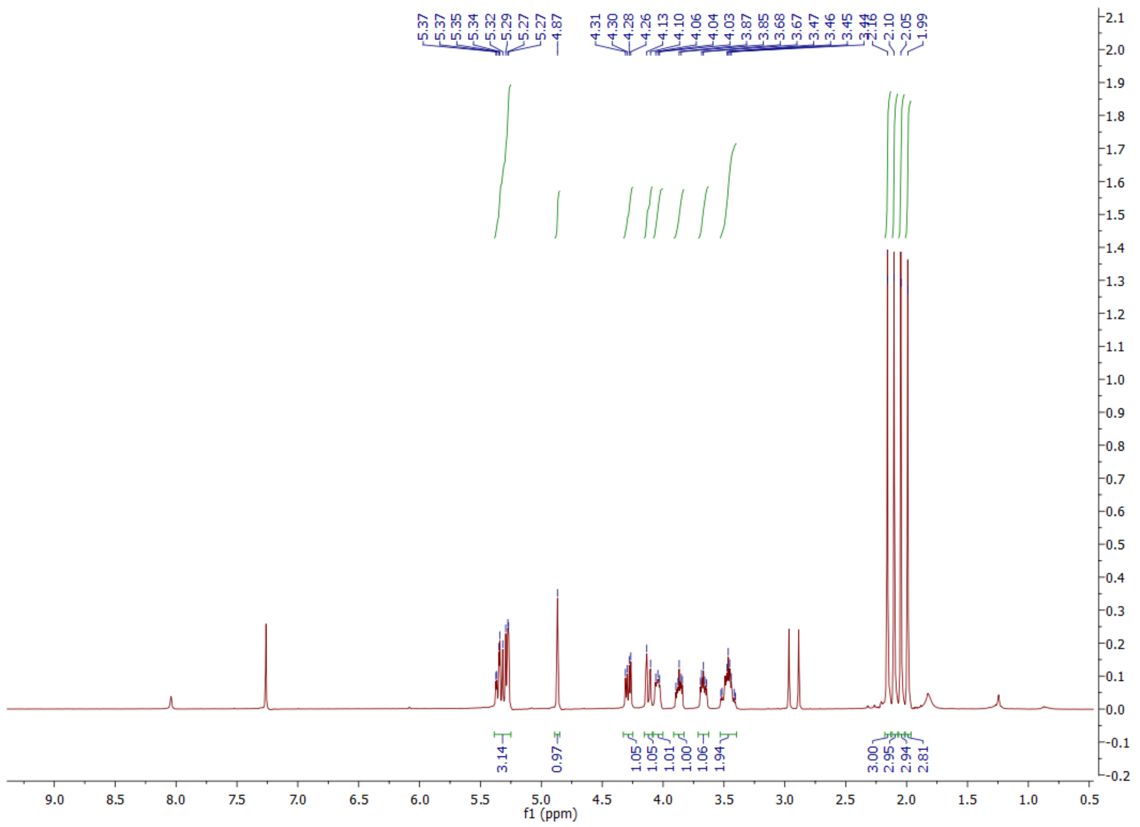

Figure S3. ^{1}H-NMR spectrum of 2-azidoethyl 2,3,4,6-tetra-O-acetyl-α-D-mannopyranoside.

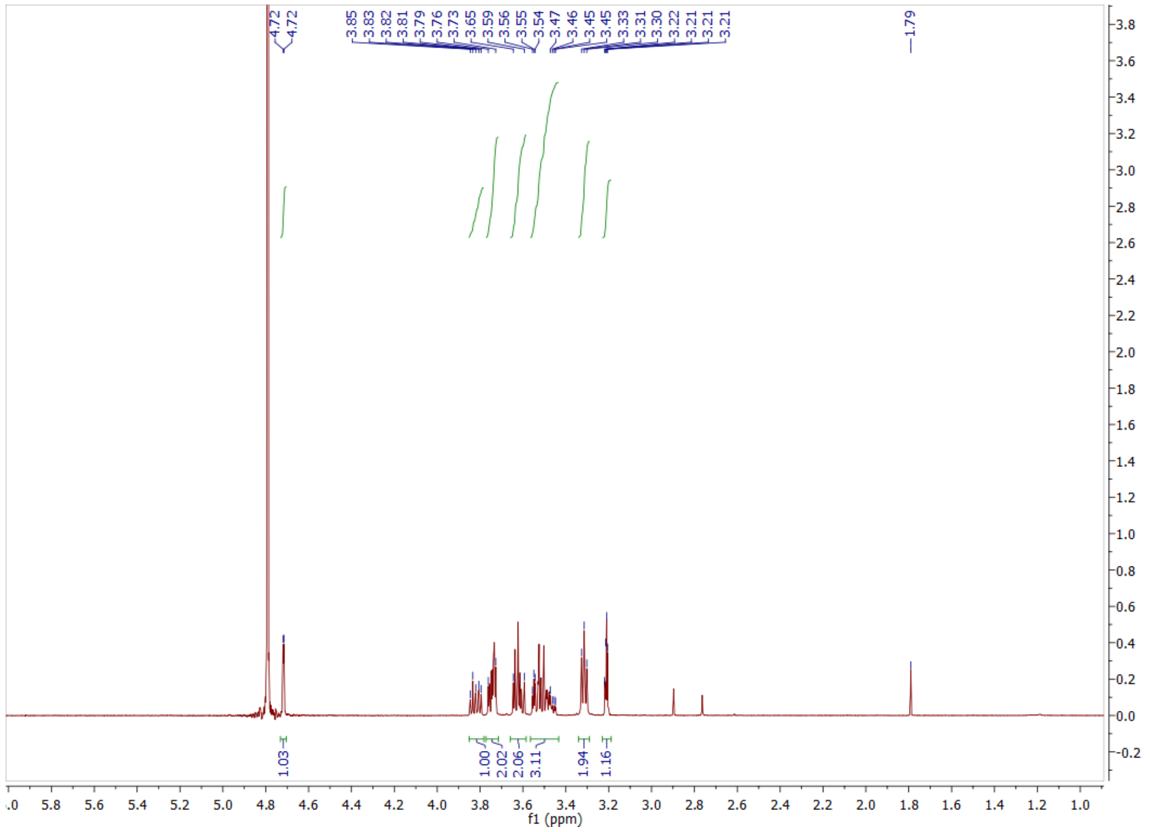

Figure S4. ^{1}H-NMR spectrum of 2-azidoethyl-α-D-mannopyranoside (Mannose-N_3).

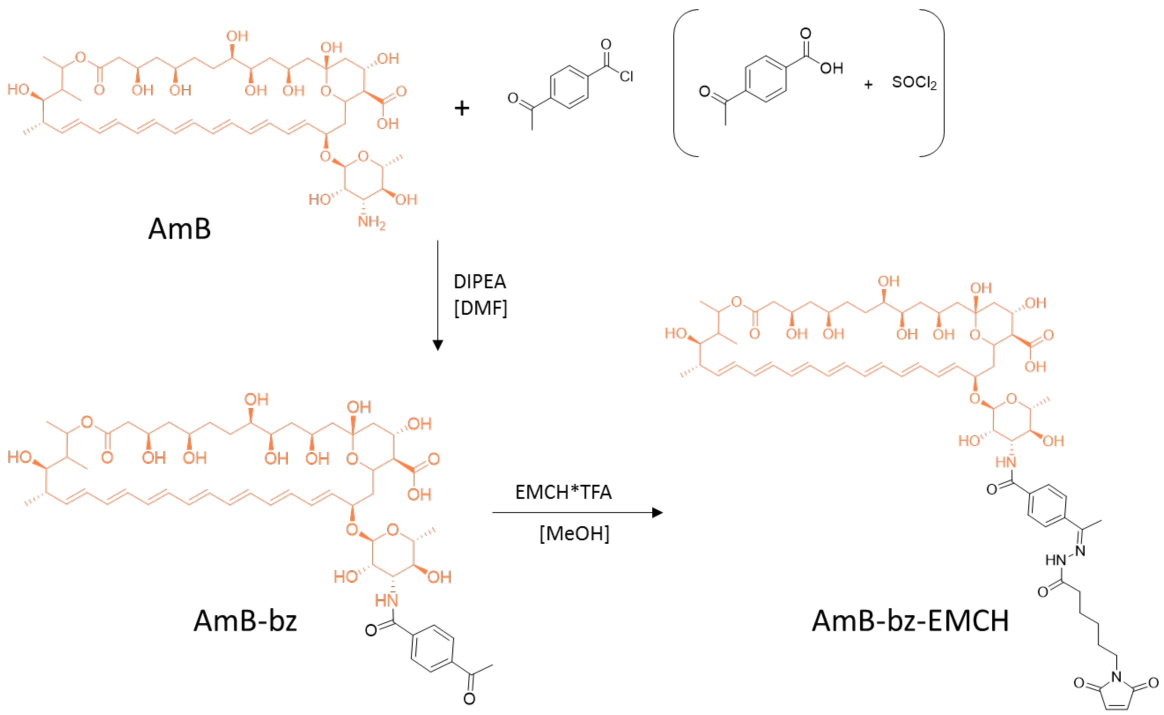

Scheme S2. Synthesis of AmB-EMCH.

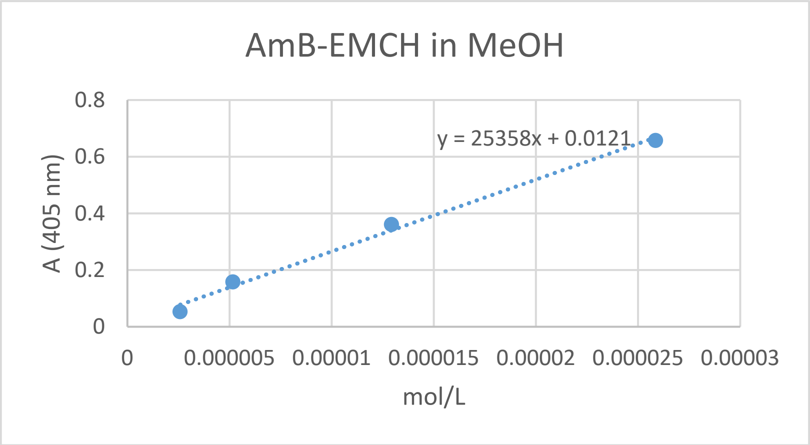

Figure S5. Calibration curve of AmB-EMCH in methanol measured by UV/Vis at 405 nm.

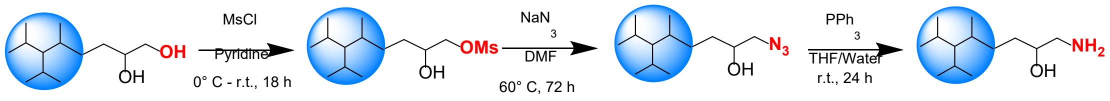

Scheme S3. Synthesis of amine-bearing polyglycerol.

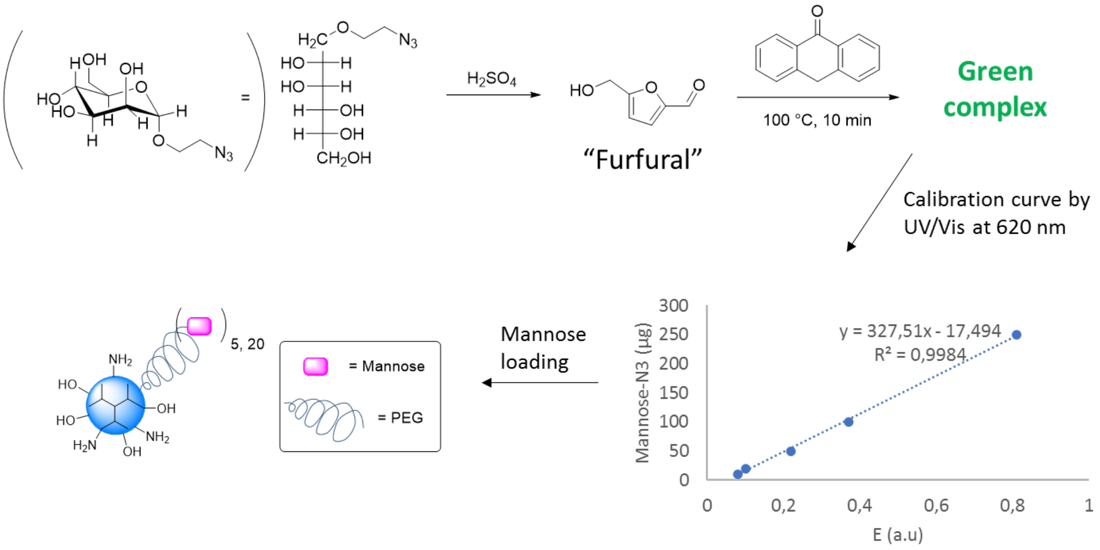

Figure S6. Quantification of mannose loading via Anthrone method.

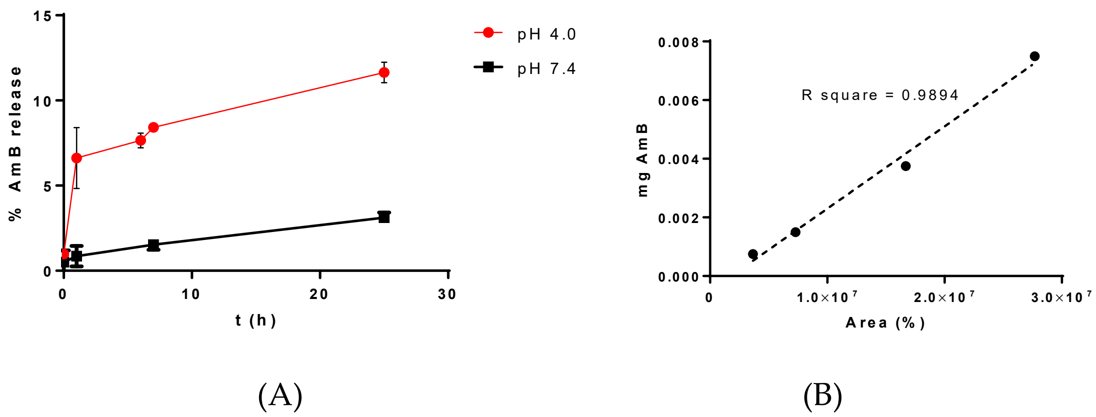

Figure S7. (**A**) Representative release profile of PG-AmB-PEG incubated at pH 4.0 and 7.4 at 37 °C over 25 h. The AmB release (%) was quantified by RP-HPLC. Mean ± SEM were obtained from triplicates in three independent experiments. (**B**) Calibration curve for AmB in methanol measured by RP-HPLC at a retention time of 1.1 min with methanol-0.005M EDTA (90:10) as mobile phase at a flow rate of 1.0 mL min^{-1} under isocratic regime. The injection volume was 25 µL.

(A)

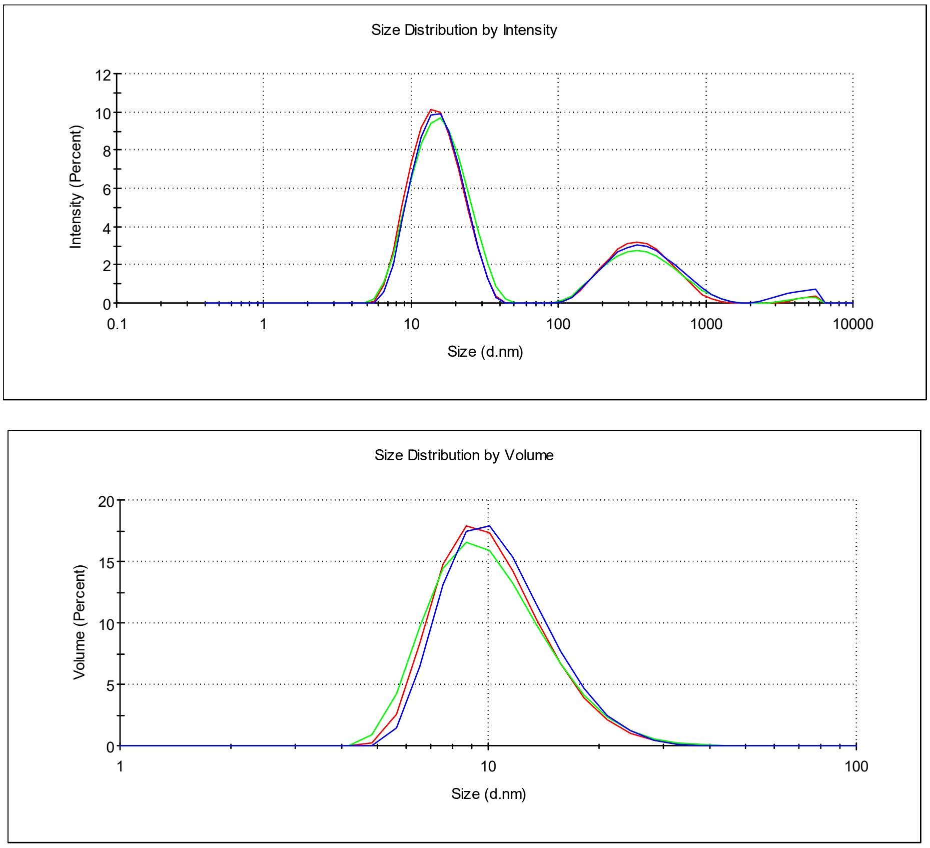
Size Distribution by Intensity
Intensity (Percent)
0
2
4
6
8
10
12
0.1
1
10
100
1000
10000
Size (d.nm)
Size Distribution by Volume
Volume (Percent)
0
5
10
15
20
1
10
100
Size (d.nm)

(B)

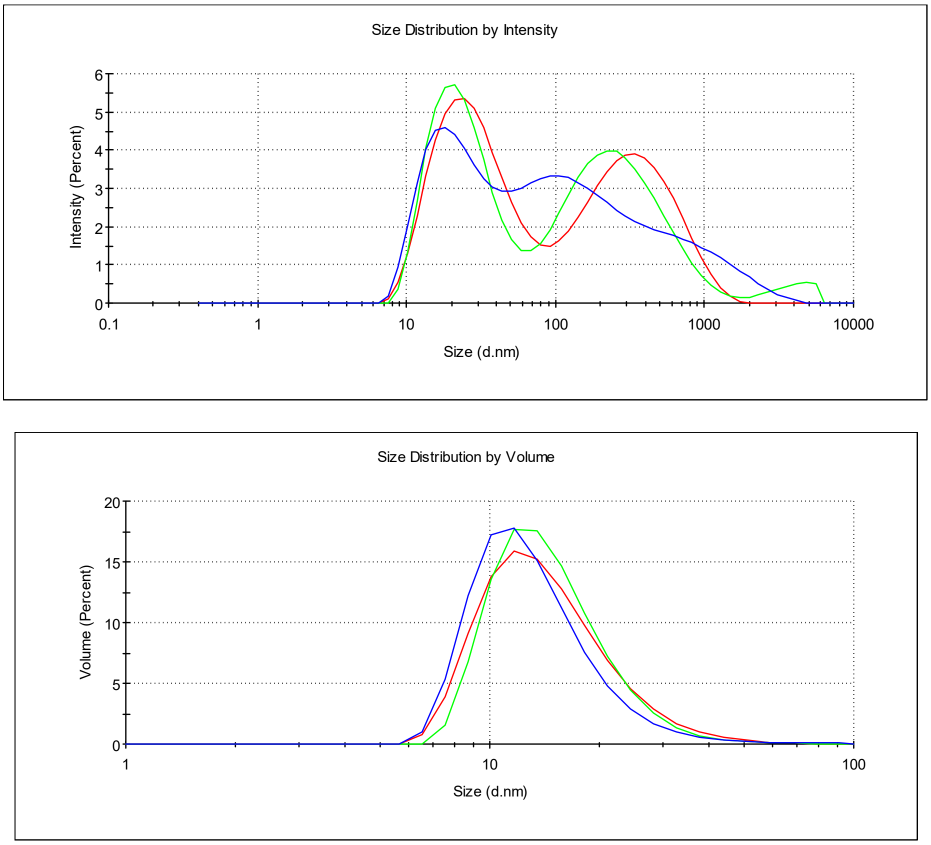
Size Distribution by Intensity
Intensity (Percent)
0
1
2
3
4
5
6
0.1
1
10
100
1000
10000
Size (d.nm)
Size Distribution by Volume
Volume (Percent)
0
5
10
15
20
1
10
100
Size (d.nm)

(C)

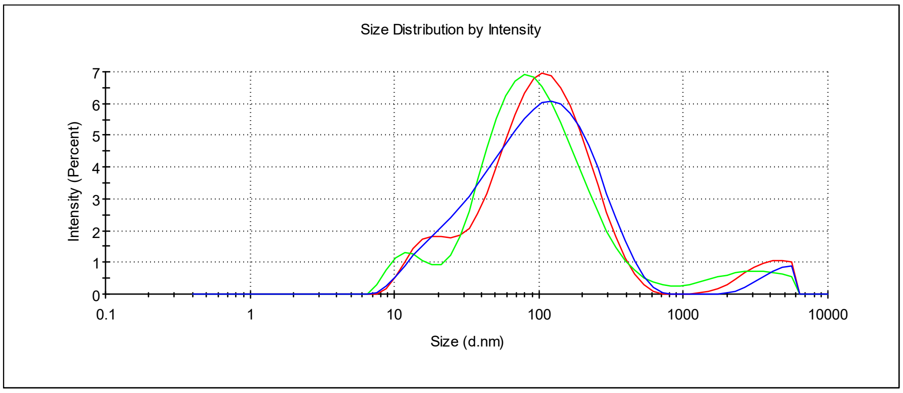
Size Distribution by Intensity
Intensity (Percent)
0
1
2
3
4
5
6
7
0.1
1
10
100
1000
10000
Size (d.nm)

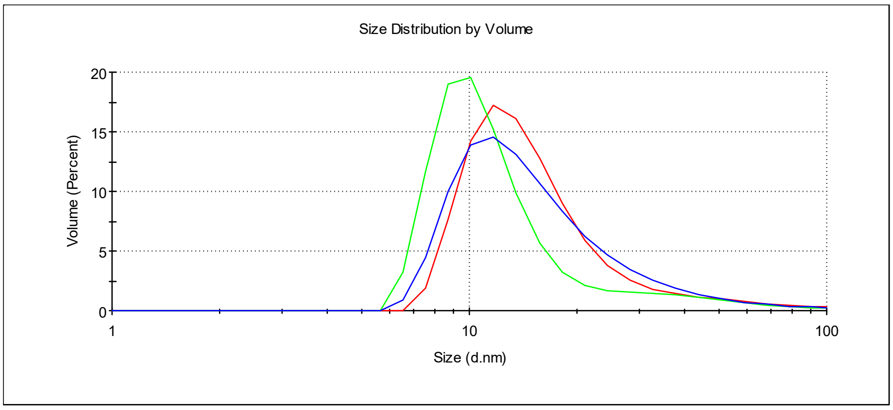
Size Distribution by Volume
Volume (Percent)
0
5
10
15
20
1
10
100
Size (d.nm)

(D)

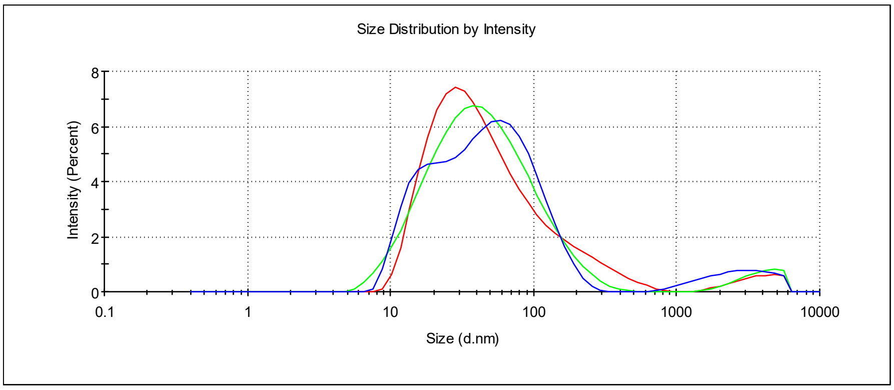
Size Distribution by Intensity
Intensity (Percent)
0
2
4
6
8
0.1
1
10
100
1000
10000
Size (d.nm)

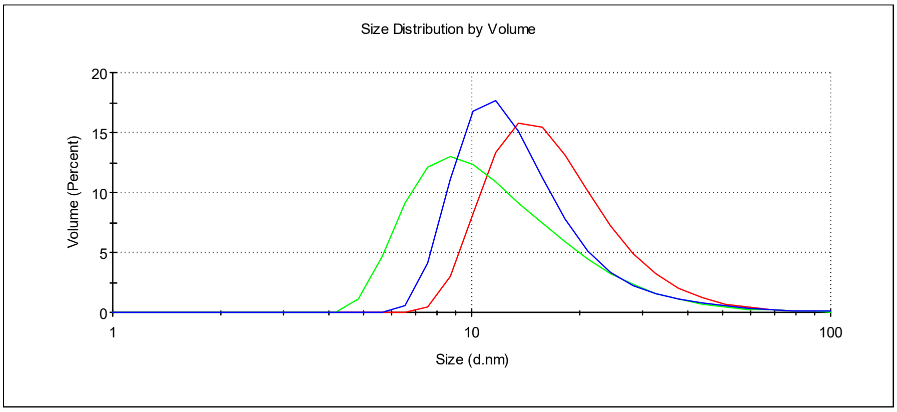
Size Distribution by Volume
Volume (Percent)
0
5
10
15
20
1
10
100
Size (d.nm)

(E)

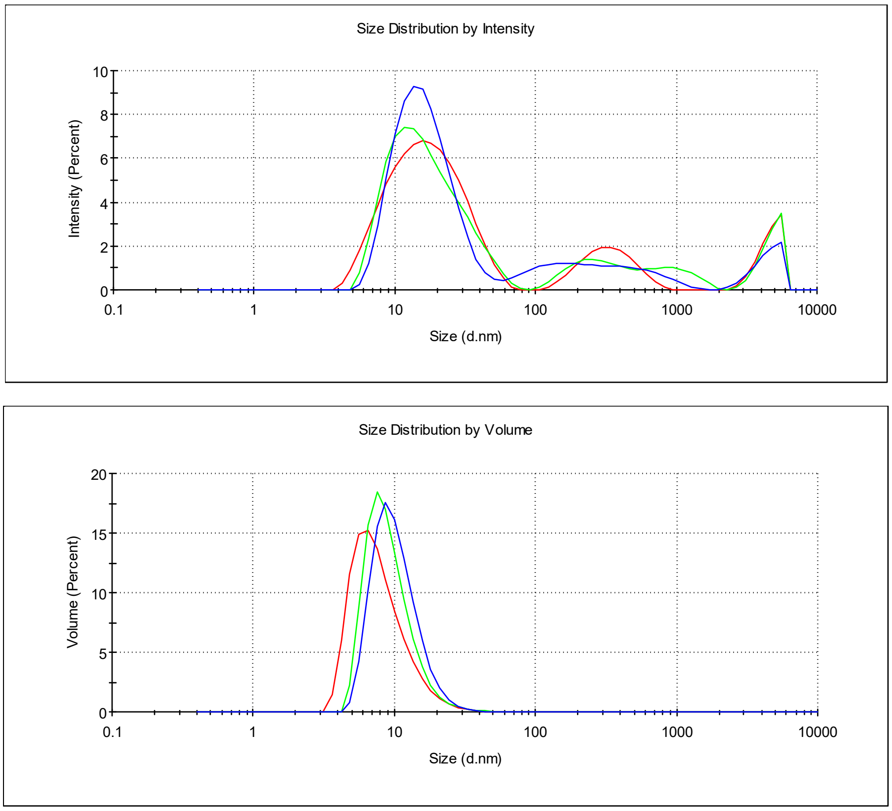

Figure S8. Dynamic light scattering measurement showing the size distribution by volume of conjugate (A) PG-PEG-FITC, (B) PG-PEG-Mann5-FITC, (C) PG-PEG-Mann10-FITC, (D) PG-PEG-Mann20-FITC and (E) PG-AmB-PEG-Mann5.

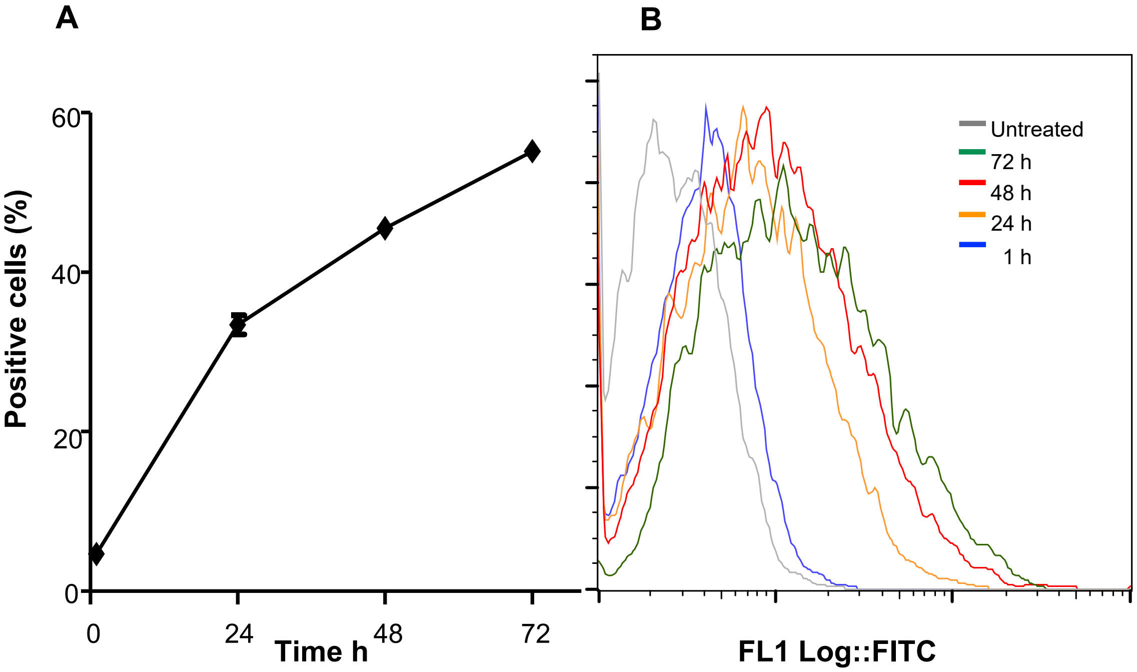

Figure S9. Cellular uptake kinetic profile obtained by flow cytometry for PG-PEG-Mann5-FITC.

© 2020 by the authors. Submitted for possible open access publication under the terms and conditions of the Creative Commons Attribution (CC BY) license (http://creativecommons.org/licenses/by/4.0/).
